# New bis-piperazine derivatives: synthesis, characterization (IR, NMR), gamma-ray absorption, antimicrobial activity, molecular docking and dynamics study

**DOI:** 10.55730/1300-0527.3767

**Published:** 2025-08-16

**Authors:** Yasemin ÜNVER, Arzu COŞKUN, Fatih ÇELİK, Halil İbrahim GÜLER, Kadriye İNAN BEKTAŞ

**Affiliations:** 1 Department of Chemistry, Faculty of Sciences, Karadeniz Technical University, Trabzon, Turkiye; 2 VocationalSchool of Health Services Department, Toros University, Mersin, Turkiye; 3 Department of Molecular Biology and Genetics, Faculty of Science, Karadeniz Technical University, Trabzon, Turkiye

**Keywords:** Piperazine, IR-NMR, gamma-ray absorption, antimicrobial, molecular dynamics, in silico study

## Abstract

A series of novel bis-piperazine derivatives (2a–2f) were synthesized and structurally characterized via Fourier transform-infrared and nuclear magnetic resonance spectroscopic techniques. Their gamma-ray shielding efficiencies were investigated through simulations on the Monte Carlo-based Geant4-GATE platform, and the results were benchmarked against data obtained from the XCOM and Phy-X software. A simulation model incorporating an NaI scintillation detector and a point gamma source was developed. Key shielding parameters, including mass attenuation coefficient, linear attenuation coefficient, half-value layer, and mean free path (MFP), were evaluated at gamma energies of 80, 120, 662, 1173, and 1332 keV. Additionally, the energy absorption buildup factor was calculated using EpiXS software, and penetration depths were assessed in the 0.015–15 MeV energy range for 10, 20, and 40 MFP values. Among the synthesized compounds, compound 2f (C_35_H_54_N_6_O_2_) had the highest gamma attenuation performance. The antimicrobial potential of compounds 2a–2f was evaluated in vitro against various microbial strains, including Gram-positive and Gram-negative bacteria as well as a fungal species. Furthermore, in silico molecular docking studies targeting DNA gyrase and GlcN-6-P synthase were performed for compounds 2d and 2f. Docking results indicated significant interactions, supporting their potential as antimicrobial agents. To assess the dynamic stability and binding persistence of the top-scoring complex (2VF5–2d), a 100 ns molecular dynamics simulation was conducted. The complex remained structurally stable throughout the trajectory, and binding free energy calculated via MM/PBSA (ΔG_bind_ = −27.31 kcal/mol) further supported the strong and favorable interaction. These results highlight compound 2d as a promising candidate for further antibacterial development.

## 1. Introduction

The piperazine ring is an important heterocyclic compound commonly found in natural products and biologically active compounds [1]. Piperazine-containing compounds have antitumor [2], antibacterial [3], antiinflammatory [4], antipsychotic [5,6], anti-Alzheimer [7], antifungal [8], and antidiabetic [9] activities. The N-4 nitrogen of piperazine can be used as a basic amine, while the N-1 nitrogen can easily incorporate hydrogen bond (H bond) acceptors and hydrophobic groups through other heterocycles without requiring the addition of a stereocenter [10]. The piperazine nucleus has 2 primary nitrogen atoms that improve the pharmacokinetic properties of drug candidates. These nitrogen sites play an important role in bioavailability, leading to a significant increase in the water solubility of drug-like molecules. Maintaining a balance between the pharmacodynamic and pharmacokinetic profiles of drug-like molecules is important for designing and developing new drugs. Thus, a key goal in the drug discovery process is to design molecules with high affinity to their targets and appropriate physicochemical properties. To this end, the piperazine template is a useful molecular subunit in rational drug design [11].

Organic compounds containing an azomethine group (–CH=N) are called Schiff bases. Schiff bases have antitumor, antiinflammatory, antibacterial, antifungal, and antipyretic activities, and are important intermediates in most enzymatic processes [12–17].

Gamma radiation is used in the nuclear industry, radiation technology, medical diagnosis and treatment, and scientific research. Therefore, gamma-ray shielding is significant for human health and the environment. The effects of ionizing radiation occur in 2 ways, depending on the exposure time and energy. While deterministic effects occur at high doses over a short period of time, long-term stochastic effects can at low radiation doses, such as secondary cancers or heart diseases [18,19]. Protecting radiosensitive organs such as the eyes, thyroid, and breast is significant in the diagnostic application of X-rays [20]. When exposure time and energy cannot be changed, the most critical protection method is to place shielding material between the source and the organism. This radiation shielding method aims to prevent harmful health problems [21,22], especially DNA mutations or skin burns as the result of photon energies in areas not covered by the shielding material. Therefore, it has become necessary to use shielding materials in areas where ionizing radiation is used to prevent many health problems [23].

Many researchers worldwide have conducted experimental, theoretical, and simulation studies to determine the radiation shielding properties of various materials [24–27]. Many materials are used for shielding purposes. Hydrogen-containing materials with low atomic weight weaken neutrons and uncharged particles due to the high cross-section of hydrogen. The addition of high atomic number materials to polymers containing a high number of H atoms is also useful for shielding gamma and X-rays [28,29]. Many studies have been examined the interaction of polymers with gamma rays and neutrons [30–39]. Photon energy is essential in the interaction of radiation with matter, where Compton scattering, Rayleigh scattering, atomic photoelectric effects, and pair production occur when photon energies are below 1000 keV.

In the present study, the gamma-ray shielding properties of polymeric materials were examined at 80, 120, 662, 1173, and 1332 keV. All 3 interactions can be observed in the selected energy ranges. The mass attenuation coefficient (MAC) of the synthesized materials was calculated, and the linear attenuation coefficient (LAC), half-value thickness (HVL), mean free path (MFP) parameters, energy absorption accumulation factor (EABF), and energy accumulation factor (EBF) were theoretically calculated in the energy range of 15–15,000 keV. MAC evaluates the interaction of the material with radiation, depending on the density of the material. Thus, the attenuation capabilities of different materials can be compared and optimized. LAC is obtained by multiplying MAC by its density. LAC is used to determine the radiation attenuation performance of the material as the number of layers increases.This parameter is critical for evaluating whether the material is effective, especially in shielding applications. HVL helps to determine how thick the material should be; therefore, it is an important reference point in practical shielding designs and safety standards. The HVL value is calculated with the formula 0.693/ LAC. MFP is important in understanding the internal structural properties of the material and the radiation interactions at the micro level. The MFP value is calculated as 1/LAC. Thus, the penetration depth of radiation can be predicted for the material. EABF and EBF measure the performance of the material at different energy levels, especially in wide energy ranges (15–15,000 keV). The effects of both low and high-energy radiation on the material can be theoretically modeled and optimized. Calculating these parameters is critical to understand and evaluate how effective the material will be against various types of radiation (gamma, X-rays, and neutrons). Theoretical calculations between 15 and 15,000 keV allow the shielding performance of the material to be predicted and optimized in different clinical and industrial applications. Safe, effective, and efficient radiation protection can be developed to minimize the risk of exposure to both patients and healthcare professionals. Antimicrobial activity has become a critical area of research in recent years due to the increasing prevalence of antibiotic-resistant pathogens and the urgent need for new therapeutic agents [40].

The increase in resistant strains of Gram-positive and Gram-negative bacteria, alongside opportunistic fungal infections, is a global health challenge that current antibiotics often fail to address. Developing new compounds with potent and broad-spectrum antimicrobial activity is essential to combat these emerging threats. Organic synthesis offers a promising avenue for the discovery of novel antimicrobial agents, capable of targeting resistant microbes and filling the gap left by traditional treatments. Investigating the antimicrobial properties of newly synthesized compounds is, therefore, a vital step toward identifying candidates with the potential to serve as effective therapeutic agents [40]. Recent research has highlighted that the antimicrobial activity of piperazine derivatives is highly influenced by the nature and position of substituents on the piperazine ring or on adjacent linker groups. For example, hydrophobic alkyl chains or electron-donating groups can improve membrane permeability and enhance interactions with bacterial targets [41–44]. Additionally, changes in the length and flexibility of linker chains such as alkyloxy bridges affect spatial orientation and lipophilic interactions with microbial enzymes [45,46]. Therefore, in our study, the systematic variation in alkyloxy bridge length across compounds 2a–2f was expected to modulate their antimicrobial potential. This hypothesis is supported by structure–activity relationship (SAR) trends reported in the literature.

In this study, new piperazin compounds 2a–2f, were synthesized and prepared and their gamma shielding parameters were examined in XCOM, GATE, and Phy-X software. The antimicrobial activity of the synthesized compounds 2a–2f was evaluated. To further understand their biological potential, a DFT study was performed to investigate the details of their molecular structures, and molecular docking studies were conducted to assess their potential interactions with bacterial target proteins. DNA gyrase and glucosamine-6-phosphate (GlcN-6-P) synthase were selected as targets due to their essential roles in bacterial survival. DNA gyrase is a key enzyme in bacterial DNA replication, while GlcN-6-P synthase plays a crucial role in peptidoglycan biosynthesis, making them well-established antibacterial targets. The docking studies provided insights into the possible binding interactions between the synthesized compounds and these bacterial enzymes, supporting their antimicrobial activity and aiding in the identification of promising lead compounds.

## 2. Materials and methods

### 2.1. Synthesis of compounds 2a–2f

To synthesize the target compounds 2a–2f, an ethanolic solution (50 mL) of 4,4’-(alkyloxy)dibenzaldehyde derivatives 1a–1f (0.01 mol) was combined with 2-(piperazine-1-yl)ethan-1-amine (0.02 mol) in a round-bottom flask. The reaction mixture was subjected to reflux under stirring for approximately 5–6 h. Reaction progress was monitored via thin-layer chromatography. Upon completion, the reaction solution was allowed to cool to room temperature, resulting in the precipitation of the target product. The crude solid was collected by vacuum filtration and purified through recrystallization using a solvent mixture of ethanol and diethyl ether in a 3:2 ratio (Scheme). The starting aldehydes 1a–1f used in this synthesis were known from the previous literature and were prepared according to established methods [47].

(1*E*,1’*E*)-1,1’-((butane-1,4-diylbis(oxy))bis(4,1-phenylene))bis(N-(2-(piperazine-1-yl)ethyl)methanimine) (2a): yield: 86.78%, mp: 118–119°C. IR (KBr, cm^−1^): 3370 (–NH), 2937 (–CH), 1640 (C=N), 1604 (C=C), 1242 (C–O–C), 831 (1,4-disubs.benzene); ^1^H NMR (400 MHz, DMSO-d_6_) δ: butyl H [1.89 (bs, 2CH_2_, 4H), 4.09 (bs, 2OCH_2_,4H)], piperazine H [2.39 (bs, 4CH_2_, 8H), 2.71 (bs, 4CH_2_, 8H)], ethyl H [2.32 (bs, 2CH_2_, 4H), 3.64 (bs, 2CH_2_, 4H)], aromatic H [6.98 (d, 4H, 8.0 Hz), 7.63 (d, 4H, 8.0 Hz)], 8.25 (s, N=CH, 2H); ^13^C NMR (100 Hz, DMSO-d_6_) δ: butyl C [25.69 (CH_2_), 67.73 (O–CH_2_)], piperazine C [45.70 (CH_2_), 58.26 (CH_2_)], ethyl C [54.30 (CH_2_), 59.71(CH_2_)], aromatic C [114.98 (4CH), 129.31 (2C), 129.86 (4CH), 160.96 (2C)], 161.09 (N=CH).

(1*E*,1’*E*)-1,1’-((pentane-1,5-diylbis(oxy))bis(4,1-phenylene))bis(N-(2-(piperazine-1-yl)ethyl)methanimine) (2b): yield: 88.90%, mp: 123–124°C. IR (KBr, cm^−1^): 3264 (–NH), 2936 (–CH), 1637 (C=N), 1604 (C=C), 1243 (C–O–C), 830 (1,4-disubs.benzene); ^1^H NMR (400 MHz, DMSO-d_6_) δ: pentyl H [1.54–1.61 (m, CH_2_, 2H), 1.77–1.82 (m, 2CH_2_, 4H), 4.04 (bs, 2OCH_2_,4H)], piperazine H [2.38 (bs, 4CH_2_, 8H), 2.70 (bs, 4CH_2_,8H)], ethyl H [2.31 (bs, 2CH_2_, 4H), 3.65 (bs, 2CH_2_, 4H)], aromatic H [6.98 (d, 4H, 8.4 Hz), 7.64 (d, 4H, 8.4 Hz)], 8.25 (s, N=CH, 2H); ^13^C NMR (100 Hz, DMSO-d_6_) δ: pentyl C [22.65 (CH_2_), 28.80 (CH_2_), 67.99 (O–CH_2_)], piperazine C [45.80 (CH_2_), 58.34 (CH_2_)], ethyl C [54.31 (CH_2_), 59.62 (CH_2_)], aromatic C [114.85 (4CH), 129.30 (2C), 129.85 (4CH), 160.02 (2C)], 161.17 (N=CH).

(1*E*,1’*E*)-1,1’-((hexane-1,6-diylbis(oxy))bis(4,1-phenylene))bis(N-(2-(piperazine-1-yl)ethyl)methanimine) (2c): Yield: 92.45%, mp: 130–132°C. IR (KBr, cm^−1^): 3262 (–NH), 2945 (–CH), 1638 (C=N), 1605 (C=C), 1241 (C–O–C), 832 (1,4-disubs.benzene); ^1^H NMR (400 MHz, DMSO-d_6_) δ: hexyl H [1.49 (bs, 2CH_2_, 4H), 1.75 (bs, 2CH_2_, 4H), 4.02 (bs, 2OCH_2_, 4H)], piperazine H [2.38 (bs, 4CH_2_, 8H), 2.70 (bs, 4CH_2_, 8H)], ethyl H [2.31 (bs, 2CH_2_, 4H), 3.65 (bs, 2CH_2_, 4H)], aromatic H [7.04 (d, 4H, 8.0 Hz), 7.63 (d, 4H, 8.0 Hz)], 8.25 (s, N=CH, 2H); ^13^C NMR (100 Hz, DMSO-d_6_) δ: hexyl C [25.61 (CH_2_), 28.84 (CH_2_), 67.98 (O–CH_2_)], piperazine C [45.66 (CH_2_), 58.12 (CH_2_)], ethyl C [54.30 (CH_2_), 59.73(CH_2_)], aromatic C [114.94 (4CH), 129.34 (2C), 129.85 (4CH), 161.02 (2C)], 161.09 (N=CH).

(1*E*,1’*E*)-1,1’-((heptane-1,7-diylbis(oxy))bis(4,1-phenylene))bis(N-(2-(piperazine-1-yl)ethyl)methanimine) (2d): yield: 90.68%, mp: 140–142°C. IR (KBr, cm^−1^): 3263 (–NH), 2938 (–CH), 1637 (C=N), 1605 (C=C), 1240 (C–O–C), 831 (1,4-disubs.benzene); ^1^H NMR (400 MHz, DMSO-d_6_) δ: heptyl H [1.42 (bs, 3CH_2_, 6H), 1.74 (bs, 2CH_2_, 4H), 4.01 (bs, 2OCH_2_, 4H)], piperazine H [2.39 (bs, 4CH_2_, 8H), 2.71 (bs, 4CH_2_, 8H)], ethyl H [2.32 (bs, 2CH_2_, 4H), 3.64 (bs, 2CH_2_, 4H)], aromatic H [6.98 (d, 4H, 8.0 Hz), 7.63 (d, 4H, 8.0 Hz)], 8.25 (s, N=CH, 2H); ^13^C NMR (100 Hz, DMSO-d_6_) δ: heptyl C [26.06 (CH_2_), 28.49 (CH_2_), 68.08 (O–CH_2_)], piperazine C [45.66 (CH_2_), 58.64 (CH_2_)], ethyl C [54.58 (CH_2_), 59.68 (CH_2_)], aromatic C [114.95 (4CH), 128.99 (2C), 130.05 (4CH), 160.69 (2C)], 161.40 (N=CH).

(1*E*,1’*E*)-1,1’-((octane-1,8-diylbis(oxy))bis(4,1-phenylene))bis(N-(2-(piperazine-1-yl)ethyl)methanimine) (2e): yield: 94.70%, mp: 144–145°C. IR (KBr, cm^−1^): 3269 (–NH), 2938 (–CH), 1638 (C=N), 1605 (C=C), 1242 (C–O–C), 831 (1,4-disubs.benzene); ^1^H NMR (400 MHz, DMSO-d_6_) δ: octyl H [1.36–1.42 (bs, 4CH_2_, 8H), 1.73 (bs, 2CH_2_, 4H), 4.0 (t, 2OCH_2_, 4H)], piperazine H [2.35 (bs, 4CH_2_, 8H), 2.66 (bs, 4CH_2_, 8H)], ethyl H [2.25 (bs, 2CH_2_, 4H), 3.63 (t, 2CH_2_, 4H)], aromatic H [6.97 (d, 4H, 8.0 Hz), 7.63 (d, 4H, 8.0 Hz)], 8.22 (s, N=CH, 2H); ^13^C NMR (100 Hz, DMSO-d_6_) δ: hexyl C [25.94 (CH_2_), 28.85 (CH_2_), 29.31 (CH_2_), 68.03 (O–CH_2_)], piperazine C [46.09 (CH_2_), 58.46 (CH_2_)], ethyl C [54.95 (CH_2_), 59.91(CH_2_)], aromatic C [114.41 (4CH), 128.57 (2C), 129.63 (4CH), 160.46 (2C)], 161.04 (N=CH).

(1*E*,1’*E*)-1,1’-((nonane-1,9-diylbis(oxy))bis(4,1-phenylene))bis(N-(2-(piperazine-1-yl)ethyl)methanimine) (2f): yield: 85.45%, mp: 150–152°C. IR (KBr, cm^−1^): 3369 (–NH), 2936 (–CH), 1637 (C=N), 1605 (C=C), 1240 (C–O–C), 830 (1,4-disubs.benzene); ^1^H NMR (400 MHz, DMSO-d_6_) δ: nonyl H [1.33 (bs, 5CH_2_, 10H), 1.74 (bs, 2CH_2_, 4H), 3.99 (bs, 2OCH_2_, 4H)], piperazine H [2.32 (bs, 4CH_2_, 8H), 2.70 (bs, 4CH_2_, 8H)], ethyl H [2.32 (bs, 2CH_2_, 4H), 3.65 (bs, 2CH_2_, 4H)], aromatic H [6.97 (bs, 4H), 7.64 (bs, 4H)], 8.25 (s, N=CH, 2H); ^13^C NMR (100 Hz, DMSO-d_6_) δ: nonyl C [25.82 (CH_2_), 29.05 (CH_2_), 29.13 (CH_2_), 29.38 (CH_2_), 67.90 (O–CH_2_)], piperazine C [45.81 (CH_2_), 58.88 (CH_2_)], ethyl C [54.47 (CH_2_), 59.77(CH_2_)], aromatic C [114.84 (4CH), 129.30 (2C), 130.28 (4CH), 161.02 (2C)], 161.84 (N=CH).

### 2.2. Molecular docking study

The accuracy of docking results is influenced by the quality of the protein and small molecule structures utilized, as well as the parameters and algorithms used in the docking calculations [48]. In this study, molecular docking calculations were conducted using AutoDock 4.2.6 software [49] to investigate the interactions of the newly synthesized compounds 2d and 2f with enzymes critical for cell wall synthesis, protein synthesis, nucleic acid (DNA) synthesis, and metabolism. The selected enzymes included DNA gyrase A and B. These enzymes are essential proteins required for bacterial DNA replication with high efficiency and accuracy, leading to bacterial cell viability [50]. DNA gyrase catalyzes the ATP-dependent introduction of negative supercoils into DNA. This bacterial protein serves as a potential target in the development of new antibacterial compounds.

Molecular docking was performed using the crystallographic structures of *Escherichia coli* DNA gyrase (GyrB) (PDB ID: 5MMN, resolution: 1.95 Å, chain: A), DNA gyrase subunit A from *Staphylococcus aureus* (PDB ID: 5CDN, resolution: 2.79 Å, chain: A), GlcN-6-P synthase (PDB ID: 2VF5, resolution: 2.90 Å, chain: A), and beta-ketoacyl-ACP synthase III (PDB ID: 1HNJ, resolution: 1.46 Å, chain: A), retrieved from the RCSB Protein Data Bank^1^. The selection of these protein targets was supported by recent studies reporting their biological relevance and structural suitability for in silico analysis. Specifically, the enzymes have previously been used in molecular docking investigations due to their critical roles in bacterial and protozoal metabolism [51–53]. These proteins have been successfully utilized as docking targets in recent publications evaluating structurally similar thiophene and sulphonate-based compounds for their antimicrobial and antileishmanial potential [51–53].

The complex structures, including water molecules, ions, and other ligands, were initially cleaned using BIOVIA Discovery Studio Visualizer 2021 [54]. Polar hydrogen atoms and Kollman charges were then added to the receptor with MGL Tools. The ligand structures were drawn with Chemdraw and converted to .pdb files using OpenBabelGUI 2.4.1 [55]. Before docking, the ligand was optimized for molecular geometry and interactions using the MMFF94s force field in Avogadro [56]. The ligand was then converted to a pdbqt file with MGL Tools. During docking, the receptor remained rigid while the ligands were flexible. A grid was set around the active site of the receptor (60 × 60 × 60 points with 0.375 Å spacing). The Lamarckian genetic algorithm in AutoDock 4.2 was used for docking, with a population of 150, 2,500,000 energy evaluations, and 54,000 generations. Binding strength predictions for compounds 2d and 2f were based on minimum binding energy and interaction patterns. The best poses were identified by binding scores and analyzed using BIOVIA Discovery Studio Visualizer 2021.

### 2.3. Molecular dynamics simulation studies

To further validate the stability and dynamic behavior of the most favorable protein–ligand complex obtained from docking studies, a 100 ns molecular dynamics (MD) simulation was conducted using GROMACS 2023.4 [57]. The complex of ligand 2d and the 2VF5 receptor (GlcN-6-P synthase) was selected based on its superior binding affinity and interaction profile. The Amber14SB force field was applied to the protein, and ligand topologies were generated using ACPYPE based on the general amber force field (GAFF) parameters [58]. The complex was embedded in a triclinic box with a minimum distance of 1.0 nm from the edges and solvated using TIP3P water molecules. To mimic physiological ionic strength and ensure charge neutrality, 0.15 M NaCl was added using the genion module in GROMACS. Long-range electrostatic interactions were handled via the particle mesh Ewald (PME) method [59] with a cutoff of 1.2 nm for real-space calculations. All covalent bonds involving hydrogen atoms were constrained using the LINCS algorithm [60]. Energy minimization was performed using the steepest descent algorithm until the maximum force on the system was reduced below 1000 kJ/mol/nm. A 2-step equilibration protocol followed: 125 ps of constant volume (NVT) equilibration at 300 K using the V-rescale thermostat, and 125 ps of constant pressure (NPT) equilibration at 1 bar using the Parrinello–Rahman barostat. During both equilibration phases, position restraints were applied to heavy atoms of the protein and ligand.

The production phase consisted of a 100 ns unrestrained MD simulation under NPT conditions using a 2 fs integration time step. Periodic boundary conditions were applied in all directions, and coordinate data were saved every 10 ps for postsimulation analysis. Structural and dynamic parameters such as root mean square deviation (RMSD), root mean square fluctuation (RMSF), radius of gyration (Rg), solvent-accessible surface area (SASA), and H bond occupancy were analyzed. In addition, binding free energies were estimated using the MM/PBSA method via the gmx_MMPBSA tool [57], based on 1000 snapshots extracted from the last 20 ns of the MD trajectory.

## 3. Results and discussion

### 3.1. Synthesis

The IR spectra of compounds 2a–2f showed that the carbonyl group (C=O) of the starting compounds, the aldehyde compound, and the NH_2_ group of the amine compound were not observed. When the IR spectra of compounds 2a–2f were examined, the carbonyl group of the starting compounds, the aldehyde compound, and the NH_2_ group of the amine compound were not observed. ^1^H NMR signals of the N=CH group, indicating the presence of a Schiff base, were observed in the range of 8.22–8.25 ppm in the ^1^H-NMR spectra as singlets. Carbon belonging to the same group signals appeared in the range of 161.04–161.84 ppm in ^13^C-NMR spectra. The H and C signals of the alkyl and phenyl groups of structures 2a–2f were observed within the expected ranges. In conclusion, ^1^H and ^13^C NMR data support the structures of compounds 2a–2f.

### 3.2. Radiation shielding parameters

**Figure 1:**
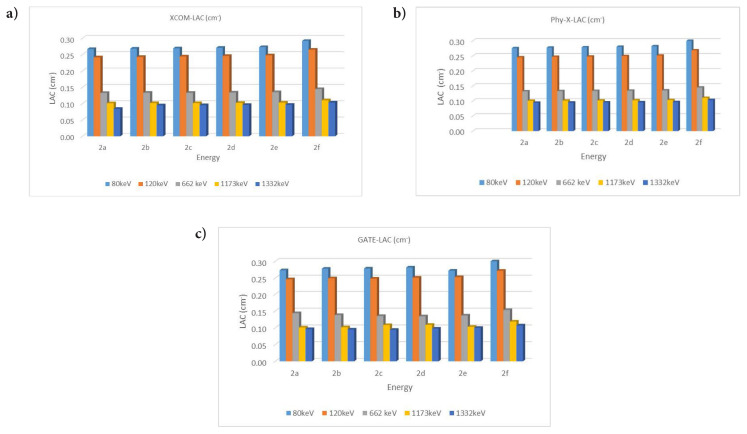
The changes in LAC values depending on photon energy, as obtained by XCOM (a), Phy-X (b), and GATE (c).

LAC was calculated from MAC (Equation 1, supplementary file). Table 1 shows the energy-dependent LAC values of all materials, which decrease with increasing energy. Figure 1 shows a graphical comparison of these values across the different materials.

**Figure 2:**
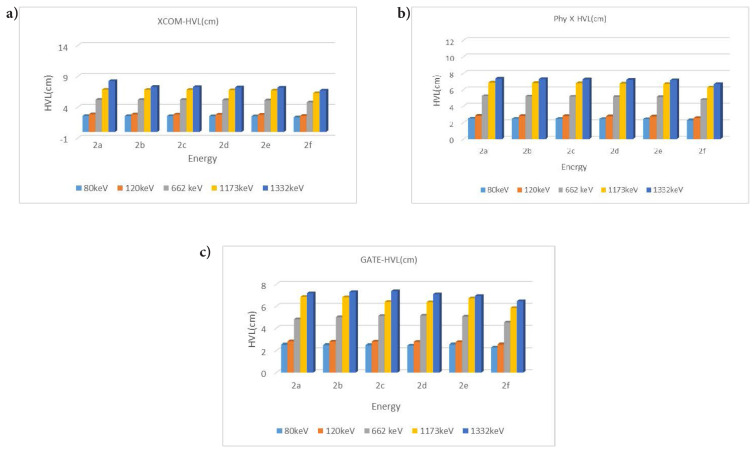
Changes in HVL depending on photon energy, as obtained by XCOM (a), Phy-X (b), and GATE (c)

**Figure 3:**
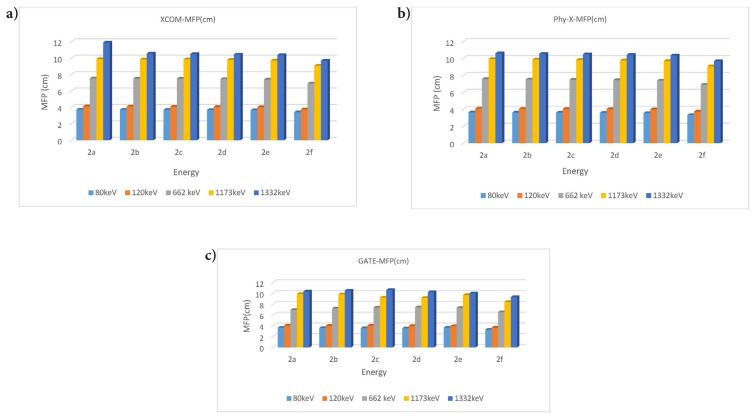
Changes in MFP depending on photon energy, as obtained by XCOM (a), Phy-X (b), and GATE (c), respectively.

HVL values were found by substituting the LAC values in Equation 2. HVL values decrease with increasing LAC values. A high HVL value indicates that the absorption feature decreases (Figures 2 and 3).

The MFP was found by substituting the LAC values in Equation 3. The obtained data are shown in Figure 3.

**Figure 4:**
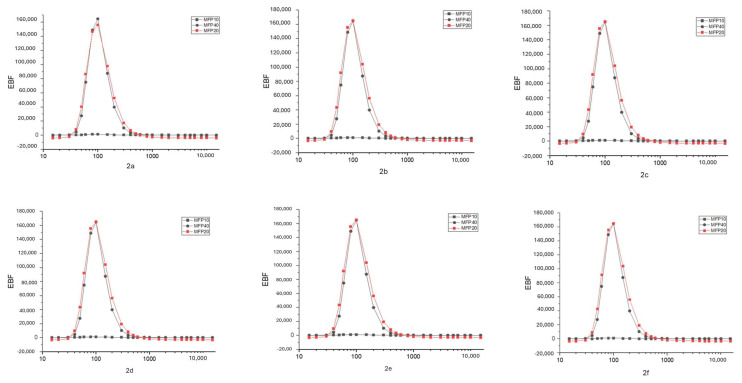
EBFs against photon energies at 10, 20, and 40 MFPs.

**Figure 5:**
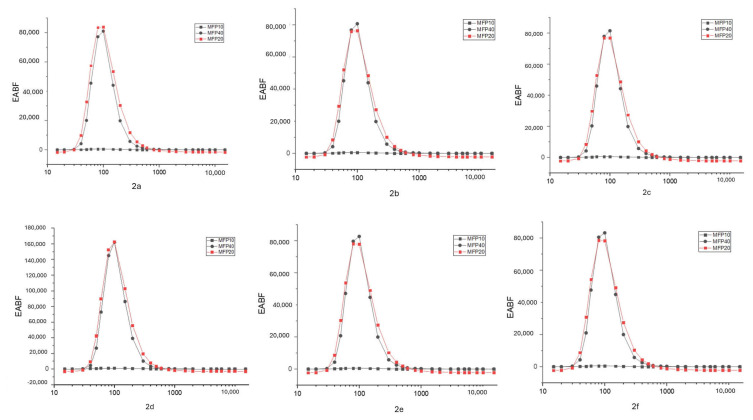
EABF against photon energies at 10, 20, and 40 MFPs.

The G-P fitting method found the EBF and EABF. It was calculated theoretically with Epi-XS up to 40 MFP thickness. EBF and EABF parameter graphs for each compound are given in Figures 4 and 5, respectively. EBF and EABF parameters are shown as energy corresponding to 10, 20, and 40 MFP values.

When gamma radiation interacts with matter at low energy with the photoelectric effect, absorption occurs. Compton scattering will be dominant with increasing energy. The scattering event in the formation of scattered photons is explained by accumulation factors (EBF, EABF). In this study, absorption and scattering parameters of gamma radiation were examined. The linear attenuation value was found by substituting the MAC value in Equation 1. The LAC value, which expresses the number of photons that pass after the incident photon intensity hits the material, showed radiation absorption. For compound 2a (C_30_H_44_N_6_O_2_), the XCOM-calculated attenuation coefficient at 80 keV is 0.2409 cm^−1^. In contrast, higher values were obtained from the Phy-X and GATE calculations, namely 0.2757 cm^−1^ and 0.2733 cm^−1^, respectively. This can be explained by the photoelectric event occurring in the interaction of the incident photon with matter. The same compound had an XCOM value of 0.0887 cm^−1^ at 1332 keV, while Phy-X and GATE values were 0.0943 cm^−1^ and 0.0964 cm^−1^, respectively. This showed that Compton scattering was dominant at high energies. The attenuation coefficients of each compound generally tend to decrease as energy increases. This is because the probability of high-energy photons interacting with the material decreases. Also, larger molecules (such as C_35_H_54_N_6_O_2_) generally had higher attenuation coefficients. The compound 2f (C_35_H_54_N_6_O_2_), which has the highest LAC value, had an XCOM value of 0.2921 cm^−1^ at 80 keV, while Phy-X and GATE values were 0.3017 cm^−1^ and 0.3027 cm^−1^, respectively. Significantly, since the increase in the number of atoms increases the density value, the LAC values increased. In contrast to the LAC value, the HVL value increased with the increase in energy, while it decreased in places where the energy was low. The HVL of all materials are given separately in Figure 2. The HVL value of compound 2f (C_35_H_54_N_6_O_2_) (with a high LAC value) was low. The MFP value obtained was lower in compound 2f with high absorption properties. All MFP values are seen in Figure 3. The values of the deposition factors depend on the depths of 10–40 mfp. For example, in the compound 2f, the EABF factor had values of 2.52, 3.19, and 3.96 at 10, 20, and 40 MFP, respectively. The EABF values of compound 2a (C_30_H_44_N_6_O_2_) (with the lowest LAC value) were calculated as 2.44, 3.06, and 3.78, respectively. The EABF factor increased with the increase in MFP. While the EBF factor was 2.43, 3.04, and 3.76 at 10, 20, and 40 MFP values for compound 2a (C_30_H_44_N_6_O_2_), these values increase to 2.50, 3.16, 3.94, respectively, for compound 2f (C_35_H_54_N_6_O_2_) (with the highest LAC value). The EABF and EBF accumulation factors decreased rapidly when the energy increased, especially after 600 keV, forming a Gaussian curve. The EABF and EBF values were minimum at 0.015 MeV in all samples. The changes in the EBF and EABF factors were seen in Figures 4 and 5, respectively. With the increase in penetration depth, secondary gamma rays (annihilation) contribute to the increase in the intensity of primary gamma rays [33].

### 3.3. Antimicrobial activity and compound-based statistical analysis

The antimicrobial activity of the synthesized compounds was tested in vitro against various microorganisms. The microorganisms tested included 4 Gram-positive bacterial species (*Bacillus cereus*, *Bacillus subtilis*, *S*. *aureus*, and *Enterococcus faecalis*), 4 Gram-negative bacterial species (*E*. *coli*, *Yersinia pseudotuberculosis*, *Pseudomonas aeruginosa*, *Klebsiella pneumoniae*), and a yeast-like fungus (*Candida albicans*).

The results are summarized in Table 2, which presents the minimum inhibitory concentrations (MIC) of the newly developed compounds, alongside the MIC values of standard antibiotics tested against the bacterial strains and yeast.

The antimicrobial activity of 6 synthesized compounds 2a–2f was evaluated and compared to a standard antibiotic across a range of microorganisms, showing notable differences in potency and spectrum of activity. Most of the compounds 2a–2c and 2e had MIC values of 625 μg/mL against the majority of bacterial strains, indicating moderate but consistent antimicrobial activity. However, compound 2e consistently showed weaker activity, with a higher MIC of 1250 μg/mL against *B*. *cereus*, *K*. *pneumoniae*, *E*. *coli*, *S*. *aureus*, and *C*. *albicans*, suggesting that 2e is less effective across both Gram-positive and Gram-negative bacteria, as well as the yeast strain.

In contrast, compounds 2d and 2f had enhanced activity against specific strains. Compound 2d was notably more effective against *B*. *subtilis*, *K*. *pneumoniae*, and *C*. *albicans*, with MIC values of 312, 312, and 78 μg/mL, respectively. Similarly, compound 2f showed significant potency against *K*. *pneumoniae* (MIC = 156 μg/mL) and *C*. *albicans* (MIC = 78 μg/mL), indicating that it had the broadest range of activity and was the most promising among the synthesized compounds. When compared to the standard antibiotic, all of the synthesized compounds had considerably higher MIC values, underscoring the superior efficacy of the antibiotic. Notably, against *P*. *aeruginosa*, where the antibiotic had a MIC of 128 μg/mL, all the compounds showed a MIC of 625 μg/mL, indicating the limited effectiveness of the synthesized compounds against this strain. However, against *C*. *albicans*, compounds 2d and 2f (MIC = 78 μg/mL) had activity comparable to that of the antibiotic (MIC = 8 μg/mL), suggesting their potential as antifungal agents. To further substantiate these findings, statistical analyses were performed to determine significant differences among the compounds. A 1-way ANOVA showed statistically significant differences in the antimicrobial effects of the compounds (p < 0.05). Tukey’s posthoc test was subsequently applied to determine pairwise differences. The results indicated that the MIC values of compounds 2d and 2f were significantly lower than those of compounds 2a, 2b, 2c, and 2e (p < 0.05), confirming their superior antimicrobial activity. Additionally, compound 2e had significantly weaker activity compared to all other compounds, with significantly higher MIC values against multiple strains (p < 0.05). In contrast, no significant differences were observed among compounds 2a, 2b, and 2c, indicating their comparable levels of antimicrobial activity. Moreover, statistical analysis of MIC values against *C*. *albicans* showed that compound 2f had significantly lower MIC values than compounds 2a, 2b, 2c, and 2e (p < 0.05), reinforcing its potential as an antifungal agent.

Overall, compounds 2d and 2f were the most effective, particularly against *B*. *subtilis*, *K*. *pneumoniae*, *S*. *aureus*, and *C*. *albicans*, while compound 2e had the least antimicrobial effect. The statistical analyses further corroborated these findings, supporting the conclusion that compounds 2d and 2f are the most promising lead compounds due to their broad-spectrum efficacy, particularly against *C*. *albicans*.

The variations in MIC values among compounds 2a–2f can be attributed to subtle structural modifications, particularly the increasing length of the aliphatic linker between aromatic Schiff base fragments. Notably, compound 2f, bearing a nonyl linker, had the strongest antimicrobial effect against both *K*. *pneumoniae* and *C*. *albicans*, likely due to improved hydrophobic interactions and enhanced membrane affinity. Similar findings were reported by Ji et al. [44] and Filipova et al. [45], where the extension of alkyl side chains or the presence of hydrophobic substituents significantly enhanced the antibacterial efficacy of piperazine-based scaffolds. These findings support the existence of a structure–activity relationship within the synthesized series and justify further modification to optimize biological performance.

### 3.4. Molecular docking

**Figure 6:**
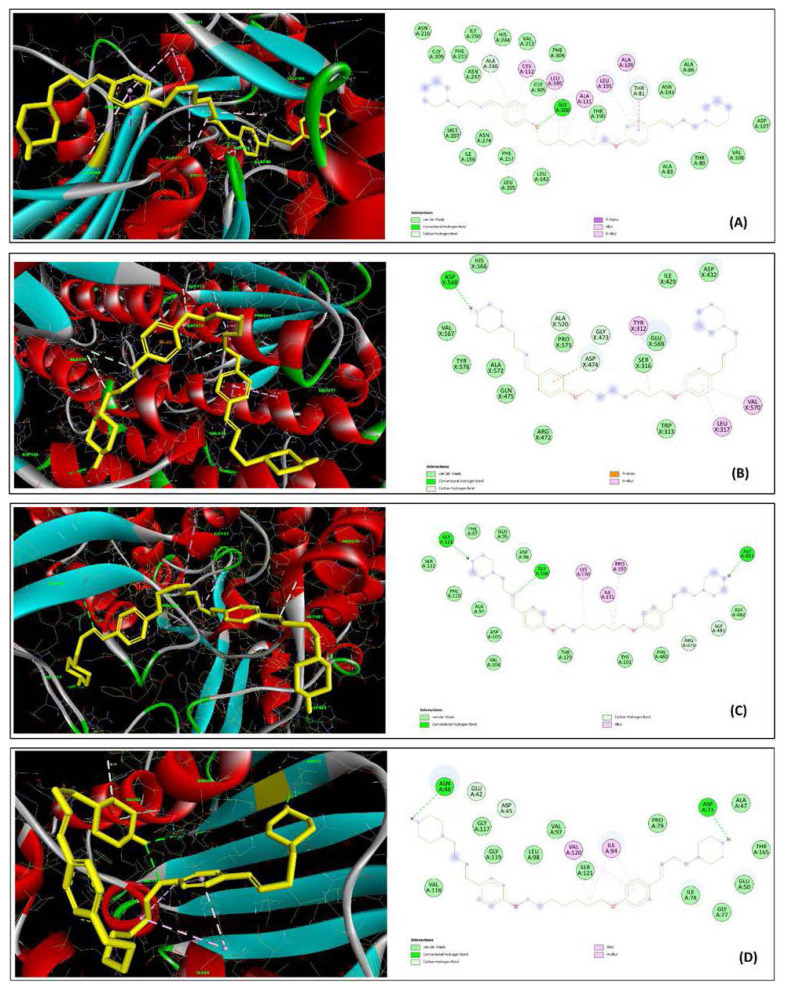
Docked poses of compound 2d in 2D and 3D representations highlighting key residues in the binding pockets of 1HJN (A), 2VF5 (B), 5CDN (C), and 5MMN (D).

**Figure 7:**
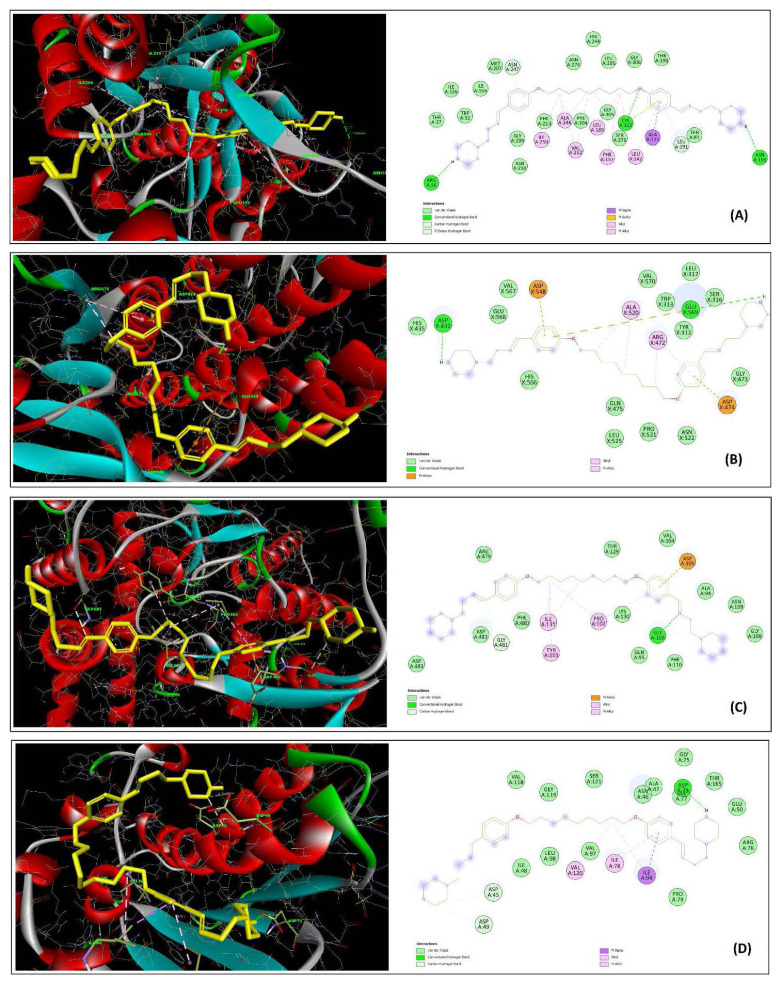
Docked poses of compound 2f in 2D and 3D representations highlighting key residues in the binding pockets of 1HJN (A), 2VF5 (B), 5CDN (C), and 5MMN (D).

Molecular docking is a critical technique for elucidating diverse binding interactions between potential drugs and specific sites or active regions on target molecules [61,62]. This approach allows for the prediction of new material properties from first principles, significantly reducing the costs and time associated with experimental trial-and-error methods. In this study, we used 2 newly synthesized molecules with extensive biological activity to investigate its potential interactions with target proteins through advanced in silico analysis. Successful docking simulations showed that both ligands, 2d and 2f, had strong binding affinities to the receptors, ranging from −7.51 kcal/mol to −10.90 kcal/mol. This range underscores the potential effectiveness of these molecules as binding agents in targeted therapeutic applications. The two ligands, 2d and 2f, were docked with beta-ketoacyl-ACP synthase II, GlcN-6-P synthase, and DNA gyrase subunit A from *S*. *aureus*, and DNA gyrase subunit B from *E*. *coli*. Figures 4 and 5 show the energetically most favorable energy-reduced docked poses. The chosen enzymes, DNA gyrase A and B, are crucial for bacterial DNA replication, ensuring high efficiency and accuracy, which is vital for bacterial cell survival [44]. DNA gyrase facilitates the ATP-dependent introduction of negative supercoils into DNA. This bacterial protein represents a promising target for the development of novel antibacterial agents. Ligand 2d had the highest binding affinity to 2VF5 and 5MMN, with binding scores of −10.90 and −9.08 kcal/mol, respectively (Figure 6). Both compounds form H bonds with Asp73 in the 5MMN (*E*. *coli* DNA gyrase (GyrB)) interaction. Asp73 is a conserved amino acid found in the ATP binding site of *E*. *coli* DNA gyrase [63]. The strongest interaction of the 2d molecule was observed with β-GlcN-6-P synthase, which showed the lowest inhibition constant of 10.19 nM. Analysis of the interactions between the 2d molecule and the receptor showed the formation of one conventional H bond, 4 carbon H bonds, 3 pi-alkyl bonds, and one pi-anion bond; 5 of these bonds had atomic distances of less than 4 Å. The most significant one was an H bond at position Asp548, with a length of 2.12 Å (Figure 6). In the docking calculations with the 2f molecule, the strongest interaction was observed with the 2VF5 structure. Analysis of the bonds showed that the most significant interaction occurred at position Glu569, with a bond length of 1.84 Å. Additionally, 2 conventional H bonds, 3 alkyl bonds, one pi-alkyl bond, and 3 pi-anion bonds were observed between the receptor and the molecule. In conclusion, the inhibition constants (K_i_ values) and interactions of the two newly synthesized ligands with 4 different proteins were thoroughly analyzed through in silico methods. Antibiotics typically target specific cellular proteins critical for various activities, effectively disrupting these essential pathways. Consequently, in line with previous research [64–68], 4 key enzymes involved in vital metabolic pathways were selected for the current study. The analysis showed that compounds 2d and 2f effectively inhibit the target proteins, showing robust antibacterial activity. These findings underscore the high potential of compounds 2d and 2f as antibacterial agents. These findings are consistent with prior studies highlighting the antibacterial potential of heterocyclic and azo-imine-based scaffolds via in silico approaches. For instance, Yeşil et al. [69] reported that fluorinated azo-azomethine derivatives had high binding affinity (up to −11.0 kcal/mol) against key microbial enzymes, supporting their potential as drug candidates. Similarly, other studies have shown that Schiff-base derivatives bearing thiophene moieties can effectively interact with targets such as DNA gyrase and GlcN-6-P synthase, aligning with the binding preferences observed in our study [70–72]. Notably, in these previous studies, compounds with structural resemblance to 2d and 2f showed significant docking scores and key H bond interactions with residues crucial to bacterial metabolism and replication. These comparative findings further validate our molecular design strategy and reinforce the pharmacological relevance of the synthesized ligands against antibacterial targets. A summary of ligand binding energies, H bonds, interacting residues, and K_i_ values is provided in Table 3. The binding poses and interactions of the ligands with the receptor proteins are illustrated in Figures 6 and 7.

### 3.5. Molecular dynamics and MM/PBSA analysis

To complement the molecular docking results and evaluate the conformational stability of the best protein–ligand complex (2VF5–2d), a 100 ns MD simulation was performed. Multiple structural and energetic descriptors were analyzed to provide a comprehensive assessment of the behavior of the system, including metrics related to overall stability, local flexibility, compactness, solvent exposure, and intermolecular interactions.

**Figure 8:**
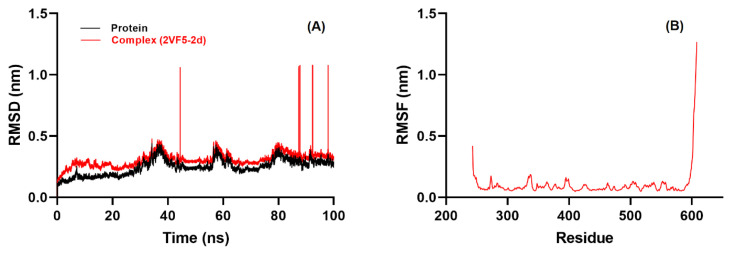
(A) RMSD profiles of free 2VF5 backbone (black) and the 2VF5–2d complex (red), showing convergence and stability after about 10 ns; (B) RMSF plot of the Cα atoms, indicating minimal fluctuations at the binding site and higher flexibility at terminal residues.

RMSD served as the primary indicator of the structural integrity of the complex over time. The 2VF5–2d complex displayed a short equilibration phase during the first ~10 ns, after which the RMSD stabilized at approximately 0.22 nm for the remainder of the simulation. This consistently low deviation suggests that the protein–ligand complex attained a stable conformation early in the simulation and remained structurally intact throughout, indicating that ligand 2d does not disrupt the native conformation of the protein backbone (Figure 8A).

RMSF analysis further corroborated structural stability by showing minimal residue-level fluctuations across most of the protein chain. The majority of residues had RMSF values below 0.15 nm, reflecting limited local flexibility (Figure 8B). Notably, residues within the ligand binding site remained particularly rigid, which supports the notion of strong and stable interactions with ligand 2d. As expected, higher fluctuations were observed at the terminal regions, which are typically more dynamic due to their solvent exposure and lack of secondary structure.

**Figure 9:**
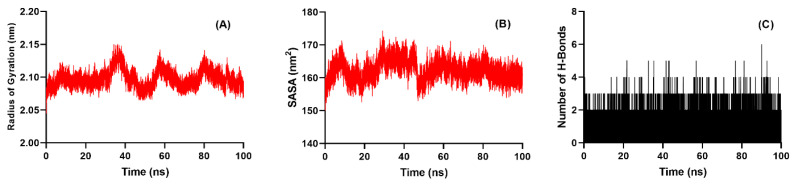
(A) Radius of gyration (Rg) of the 2VF5 protein backbone over 100 ns indicating sustained protein compactness; (B) Solvent-accessible surface area (SASA) plot showing stable solvent exposure of the protein throughout the simulation; (C) Number of intermolecular H bonds between ligand 2d and 2VF5, showing 2–4 persistent H bonds across the trajectory, which supports binding stability.

Radius of gyration (Rg) analysis was conducted to evaluate the global compactness of the protein. The Rg values fluctuated minimally around an average of 2.18 nm, suggesting that the protein preserved its folded, globular structure and did not undergo partial unfolding (Figure 9A). This finding implies that ligand binding did not induce destabilization or structural collapse, thereby supporting the conformational resilience of the protein under physiological simulation conditions.

SASA values remained largely unchanged throughout the simulation, further confirming the structural integrity of the protein. The lack of significant variations in solvent exposure—particularly in the binding region—indicates that the ligand did not trigger conformational changes that would expose or shield additional protein surface area. This stability is consistent with a compact, well-folded protein–ligand complex (Figure 9B).

H bond analysis highlighted the dynamic but persistent nature of key intermolecular interactions. Between 1 and 4 H bonds were consistently formed throughout the simulation, with an average of 2–3 stable H bonds (Figure 9C). Key interactions with residues such as Asp548 and Glu569 were maintained over time, underscoring the specificity and durability of the binding mode. These persistent interactions further validate the high docking scores previously observed and show that ligand 2d remains securely anchored within the active site.

In summary, the MD results affirm the thermodynamic and structural stability of the 2VF5–2d complex. All evaluated parameters—RMSD, RMSF, Rg, SASA, and hydrogen bonding—consistently support the conclusion that ligand 2d engages the target protein with high affinity and minimal perturbation, reinforcing its potential as a promising antibacterial agent.

### 3.6. MM/PBSA binding free energy and per-residue decomposition analysis

To further validate the molecular docking and MD simulation results, the molecular mechanics/poisson–boltzmann surface area (MM/PBSA) method was applied to estimate the binding free energy of the 2VF5–2d complex. The total binding free energy was calculated as −27.31 kcal/mol, which is indicative of a favorable and spontaneous interaction. The dominant contributions to this binding energy originated from van der Waals interactions (−42.73 kcal/mol) and electrostatic interactions (−22.59 kcal/mol), while polar solvation energy contributed unfavorably (+41.99 kcal/mol). The nonpolar solvation term also provided a stabilizing effect (−3.98 kcal/mol). These results are summarized in Table 4. These findings highlight the significant role of hydrophobic contacts and electrostatic complementarity in stabilizing the complex, consistent with the hydrogen bonding and compactness observed in the MD simulations.

To gain a more detailed understanding of key residue contributions, a per-residue decomposition analysis was performed. Among the active site residues, ARG311, GLU315, and TRP313 had the most substantial total binding energy contributions, ranging between −5.4 to −21.1 kcal/mol. In particular, ARG311 contributed −21.07 kcal/mol, which underscores its essential role in anchoring ligand 2d via electrostatic and polar interactions. Similarly, GLU315 and TRP313 had strong favorable interactions due to their strategic positioning in the binding pocket and their capacity to form H bonds and π-contacts.

These residue-level insights provide a mechanistic rationale for the strong and stable binding of compound 2d, aligning well with the molecular docking scores and dynamic stability profiles obtained in earlier stages. Overall, the MM/PBSA results offer quantitative support for the high binding affinity and pharmacological potential of ligand 2d against GlcN-6-P synthase (2VF5)

## 4. Conclusions

Compounds 2a-2f were synthesized and characterized by Fourier transform-infrared and nuclear magnetic resonance spectroscopic methods. This study investigated the changes in gamma radiation attenuation parameters by calculating the energy-dependent changes in linear attenuation coefficients. The LAC value and EBFs increased with the increase in the number of atoms in the polymer materials examined. The results of all three theoretical studies were consistent. As a result of the calculations, the highest gamma radiation attenuation property belongs to the compound 2f (C_35_H_54_N_6_O_2_). Compounds 2d and 2f were the most effective, particularly against *B*. *subtilis*, *K*. *pneumonia*, *S*. *aureus* and *C*. *albicans*, while 2e had limited antimicrobial effects. The results suggest that compounds 2d and 2f stand out as potential lead compounds due to their broad-spectrum efficacy, especially against *C*. *albicans*. The inhibition constants (K_i_ values) and interactions of the newly synthesized ligands with 4 different proteins, crucial for metabolic pathways, were thoroughly analyzed through in silico methods. Antibiotics typically target specific cellular proteins critical for various activities, effectively disrupting these essential pathways. Consequently, 4 key enzymes involved in vital metabolic pathways were selected for the current study. The analysis showed that compounds 2d and 2f effectively inhibit the target proteins, showing robust antibacterial activity. These findings underscore the high potential of compounds 2d and 2f as antibacterial agents. To further corroborate these findings, a 100 ns MD simulation was performed on the 2VF5–2d complex, which had shown the highest docking affinity. The trajectory analyses showed stable RMSD and Rg profiles, minimal fluctuations at critical binding residues (RMSF), consistent solvent exposure (SASA), and sustained intermolecular H bonds, indicating that the complex remained conformationally stable under dynamic conditions. Additionally, MM/PBSA binding free energy calculations yielded a favorable ΔG_binding_ of −27.31 ± 1.90 kcal/mol, supporting the strong and stable interaction of ligand 2d with GlcN-6-P synthase. These results provide dynamic validation of the docking predictions and further reinforce the potential of compound 2d as a structurally robust antibacterial candidate.

## Supplementary file

### 1. Instrumentation

IR spectra of the synthesized compounds were taken on a Perkin Elmer (Waltham, MA, USA) Fourier transform-infrared 1600 (4000–400 cm^−1^) spectrophotometer device, and ^1^H-NMR, ^13^C-NMR spectra were taken on a Bruker (Billerica, MA, USA) 400 MHz nuclear magnetic resonance device with DMSO-d_6_ solvent. Melting points were determined using a thermo-var apparatus fitted with a microscope and are uncorrected. The solvents and chemicals used in synthesis and structure elucidation were obtained from Fluka (Buchs, Switzerland), Merck (Darmstadt, Germany), and Sigma-Aldrich (St. Louis, MO, USA), and all solvents were subjected to appropriate purification and drying processes.

### 2. Theoretical context for gamma-ray absorption

The theoretical framework used to calculate gamma-rays shielding parameters are outlined as follows.

#### 2.1. Mean free path, linear attenuation coefficient, and half-value layer

Depending on the physical state, the absorber density of the material is proportional to the linear attenuation coefficients. However, MAC is usually used to eliminate density dependence. Thus, MAC values (cm^2^/gr) of alloys are defined as follows:


(1) $$\mu =\frac{\mu_{m}}{\rho }=\Sigma_{i}W_{i}(\mu /\rho )$$

where *ρ*, *μ*, and *wi* denote the sample density, LAC, and weight fraction, respectively, and *μ* is dependent on the incident gamma-ray energy and Z of the elements of the medium [1].

Shield materials and MFP properties are required to calculate the strength of the gamma-ray shield. HVL is the sample thickness that will reduce the intensity of the primary photon beam by half. These values can be calculated as follows [2]:


(2) HVL=ln2/μ

(3) MFP=1/μ

#### 2.2. Exposure buildup factors

The equivalent atomic number must first be calculated as the exposure accumulation factor. Zeq for a given trial was calculated by matching the ratio $\frac{\frac{\mu }{\rho }\text{Compton}}{\frac{\mu }{\rho }\text{Total}}$ of a given material at a given energy with the corresponding ratio of the material at the same energy. Thus, the Compton partial MAC, μ/ρ Compton, and the total MACs, μ/ρ Total, were first obtained for the elements Z = 4–40 and selected materials in the 0.015–15 MeV energy region using WinXCom software [3,4]. When the ratio $\frac{\frac{\mu }{\rho }\text{Compton}}{\frac{\mu }{\rho }\text{Total}}$ lies between two consecutive compound ratios, the interpolation of Zeq was calculated using the following equation:


(4) $$\text{Zeq}=\frac{\left[Z_{1}\;(\text{log }R_{2}-\text{log }R)+Z_{2}\;(\text{log }R-\text{log }R_{1})\right]}{\left(\text{log }R_{2}-\text{log }R_{1}\right)}$$

The G-P fit parameters are calculated using an interpolation procedure. The G-P fit parameters for the elements are obtained from the ANSI/ANS-6.4.3 standard reference database, which provides G-P fit parameters for elements from beryllium to iron from 0.015–15 MeV to 40 mfp. The G-P fit formulas are as follows [5]:


(5) $$B\;(E,X)=1+\frac{b-1}{K-1}(K^{x}-1)\;\;\;\text{K}\neq 1$$

(6) B (E,X)=1+(b-1)x         for, K=1

(7) $$K(E,x)=cx^{a}+d\frac{\left[\text{tanh}\left(\frac{x}{X_{k}}-2\right)-\text{tanh}(-2)\right]}{1-\text{tanh}(-2)}\;\;\;\text{For},\;x\leq 40\;mfp$$

E; is the incident photon energy,
X; penetration depth in mfp (cm),
a,b,c,d, and X_k;_ G-P fitting parameters,
b; buildup factor at one mfp,
K; photon dose multiplication and the change in the spectrum’s shape,
Mfp; A mean free path of a photon in a medium is the average distance traveled by a photon before interacting and is given as 1/μ.

#### 2.3. Monte Carlo simulations and XCOM

The radiation absorption properties of the materials were investigated using the setup prepared with a Geant4-GATE simulation. The setup by Coşkun et al. was used in previous studies [6]. The obtained values were compared with the values of XCOM software and Phy-X. The XCOM software developed by Berger and Hubbel [7] is an open-access software program that can calculate the radiation absorption properties of elements, compounds, or mixtures at selected energy values between 1 keV and 100 GeV. A few studies examine the mass absorption properties of radiation shielding materials with Geant4-based GATE (Geant4 Application for Tomographic Emission) simulation. GATE simulation is a software program in C++ that performs Monte Carlo calculations that can be used in nuclear physics, radiology and radiotherapy medical applications.

#### 2.4. Phy-X/PSD PC program

Phy-X/PSD software is a software program that can calculate photon absorption parameters. It is a software program running on a mote server. As an operating system, it runs on Ubuntu 14.04.3 LTS and Intel Core i7-2600 CPU @ 3.40 GHz, 1 GB installed memory. The programming language is NodeJS v8.4.0, offering Nginx 1.15.8. For security purposes, 256-bit Positive SSL encryption is used between the processor and the server, which is secure [8]. All users need to access https://phy-x.net/PSD to run the PSD software.

#### 2.5. EpiXS program

EpiXS [9] is a Windows based application that can be used for radiation shielding, dosimetry calculations and is based on EPICS2017 of ENDF/B-VIII and EPDL97 of ENDF/B-VI.8. The software calculates partial or total cross sections (σ), MACs (μ/ρ), linear attenuation coefficients (μ), MFP, HVL, sufficient atomic numbers (Zeff), electron densities (Neff) and accumulation factors with any EABF and EBF parameters by GP-fitting method [10].

### 3. Antimicrobial activity method

The newly synthesized compounds underwent assessment for their antimicrobial properties against nine distinct pathogenic microorganisms responsible for various diseases. This selection encompassed eight microorganisms, encompassing both Gram-negative bacteria (*E*. *coli* ATCC 25922, *Yersinia pseudotuberculosis* ATCC 911, *Pseudomonas aeruginosa* ATCC 17853, *Klebsiella pneumoniae* ATCC 700603) and Gram-positive bacteria (*S*. *aureus* ATCC 25923, *Bacillus subtilis* ATCC 6633, *Bacillus cereus* ATCC 10876, *Enterococcus faecalis* ATCC 29212), as well as yeast-like fungi (*Candida albicans* ATCC 10231, *Candida tropicalis* ATCC 13803). These microorganisms were procured from the American Type Culture Collection.

The minimal inhibitory concentration (MIC) values, expressed in micrograms per milliliter (μg/mL), of the newly synthesized compounds were ascertained utilizing the microtitre broth dilution method, accompanied by the rapid INT colorimetric assay as detailed in Kuete et al. [11], following the guidelines established by the Clinical and Laboratory Standards Institute (CLSI). To prepare stock solutions of the synthesized compounds, all compounds were dissolved in dimethyl sulfoxide (DMSO). These stock concentrations were then subjected to a 2-fold serial dilution in 96-well microplates using Mueller–Hinton broth (MHB) as the diluent. Bacterial suspensions were prepared at a concentration of approximately 5 ×10^−5^ colony-forming units (CFU) per milliliter and were subsequently inoculated into each well. The microplates were then incubated at 37 °C for a period of 24 h. After the 37 °C incubation, each well received an addition of 40 μL of a 0.2 mg/mL solution of p-iodonitrotetrazolium chloride (INT), serving as an indicator of microbial growth. The microplates were further incubated for 30 min at 37 °C, during which the MIC values were visually determined. The MIC value was recognized as the lowest concentration at which the growth of the tested microorganism was entirely inhibited, which corresponded to the appearance of the first clear well. MIC values were determined at least in triplicate, with the value being considered the final MIC when it was repeated at least two times. Ampicillin (10 mg/mL) and fluconazole (2 mg/mL) were used as standard commercial drugs for bacterial and yeast testing, respectively. A negative control experiment was conducted, using only DMSO to account for any inhibitory effects stemming from the solvent.

Figure S1 IR spectrum of compound 2a.

Figure S2 ^1^H-NMR spectrum of compound 2a.

Figure S3 ^13^C-NMR (APT) spectrum of compound 2a.

Figure S4 IR spectrum of compound 2b.

Figure S5 ^1^H-NMR spectrum of compound 2b.

Figure S6 ^13^C-NMR (APT) spectrum of compound 2b.

Figure S7 IR spectrum of compound 2c.

Figure S8 ^1^H-NMR spectrum of compound 2c.

Figure S9 ^13^C-NMR (APT) spectrum of compound 2c.

Figure S10 IR spectrum of compound 2d.

Figure S11 ^1^H-NMR spectrum of compound 2d.

Figure S12 ^13^C-NMR (APT) spectrum of compound 2d.

Figure S13 IR spectrum of compound 2e.

Figure S14 ^1^H-NMR spectrum of compound 2e.

Figure S15 ^13^C-NMR (APT) spectrum of compound 2e.

Figure S16 IR spectrum of compound 2f.

Figure S17 ^1^H-NMR spectrum of compound 2f.

Figure S18 ^13^C-NMR (APT) spectrum of compound 2f.

References1

ManoharaSR
HanagodimathSM
ThindKS
GerwardL

On the adequate atomic number and electron density: a comprehensive set of formulas for all materials and energies above one keV
Nuclear Instruments and Methods in Physics Research Section B
2008
266
18
3906
3912
10.1016/j.nimb.2008.06.034
2

El-SayedA
AliMAM
IsmailMR

Natural fiber high-density polyethylene and lead oxide composites for radiation shielding
Radiation Physics and Chemistry
2003
66
3
185
195
10.1016/S0969-806X(02)00470-X
3

GerwardL
GuilbertN
JensenKB
LevringH

X-ray absorption in matter
Reengineering XCOM, Radiation Physics and Chemistry
2001
60
1–2
23
24
10.1016/S0969-806X(00)00324-8
4

GerwardL
GuilbertN
JensenKB
LevringH

WinXCom-a program for calculating X-ray attenuation coefficients
Radiation Physics and Chemistry
2004
71
3–4
653
654
10.1016/j.radphyschem.2004.04.040
5

ManoharaSR
HanagodimathSM

Studies on adequate atomic numbers and electron densities of essential amino acids in the energy range one keV–100 GeV
Nuclear Instruments and Methods in Physics Research Section B
2007
258
2
321
328
10.1016/j.nimb.2007.02.101
6

BergerMJ
HubbellJH

XCOM: photon cross sections on a personal computer, National Bureu of Standarts Washington, DC (USA), Cent
Radiat Res
1987
1
28
10.2172/6016002
7

CoşkunA
ÇetinB
Yiğitoğluİ
TopaklıH

Comparison of PbO doped ZrB2 glasses’ radiation absorption properties using GATE-GEANT4 Monte Carlo code and XCOM program
International Journal of Computational and Experimental Science and Engineering
2023
9
3
274
279
8

SakarE
OzpolatOF
AlımB
SayyedMI
KurudirekM

Phy-X / PSD: Development of a user-friendly Online Software for Calculation of Parameters Relevant to Radiation Shielding and Dosimetry
Radiation Physics and Chemistry
2020
166
108496

https://doi.org/10.1016/j.radphyschem.2019.108496
https://www.pnri.dost.gov.ph/index.php/downloads/software/epixs

10

HilaFC
Asuncion-AstronomoA
DingleCAM
JecongJFM
Javier-HilaAMV


EpiXS: A Windows-based program for photon attenuation, dosimetry and shielding based on EPICS2017 (ENDF/B-VIII) and EPDL97 (ENDF/B-VI. 8)
Radiation Physics and Chemistry
2021
182
109331
10.1016/j.radphyschem.2020.109331
11

KueteV
BetrandteponnoR
MbavengAT

Antibacterial activities of the extracts, fractions and compounds from *Dioscorea bulbifera*
BMC Complement Altern Med
2012
6882-12-228
10.1186/1472-6882-12-228PMC352847123176193

## Figures and Tables

**Scheme:**
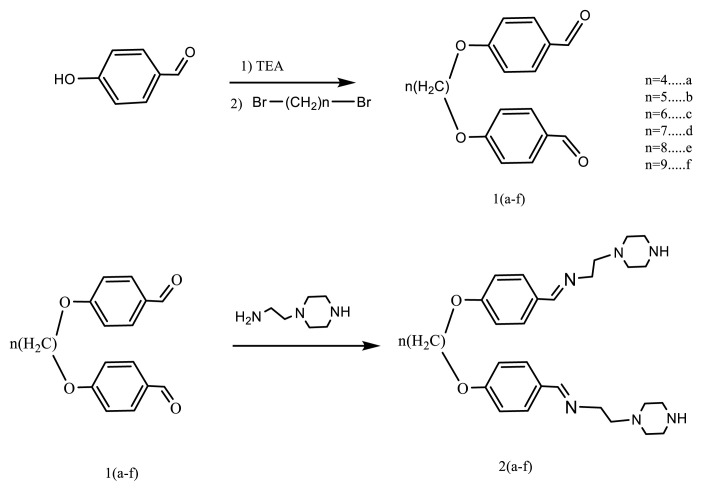
Synthesis pathway for compounds 2a–2f.

**Table 1:** LAC values.

Material		*Linear Attenuation Coefficient (cm* *^−1^* *)*				
		80keV	120keV	662 keV	1173keV	1332keV
*C_30_H_44_N_6_O_2_*(2a)	XCOM	0.2409	0.2409	0.1190	0.0911	0.0887
	Phy-X	0.2757	0.2452	0.1322	0.1007	0.0943
	GATE	0.2733	0.2465	0.1438	0.1011	0.0964
*C_31_H_46_N_6_O_2_*(2b)	XCOM	0.2684	0.2430	0.1334	0.1016	0.0948
	Phy-X	0.2774	0.2468	0.1330	0.1013	0.0949
	GATE	0.2779	0.2493	0.1382	0.1016	0.0950
*C_32_H_48_N_6_O_2_*(2c)	XCOM	0.2694	0.2445	0.1333	0.1016	0.0952
	Phy-X	0.2784	0.2477	0.1335	0.1017	0.0953
	GATE	0.2786	0.2484	0.1353	0.1083	0.0939
*C_33_H_50_N_6_O_2_*(2d)	XCOM	0.2711	0.2461	0.1341	0.1023	0.0959
	Phy-X	0.2802	0.2493	0.1344	0.1024	0.0959
	GATE	0.2817	0.2512	0.1344	0.1088	0.0976
*C_34_H_52_N_6_O_2_*(2e)	XCOM	0.2731	0.2479	0.1351	0.1030	0.0964
	Phy-X	0.2822	0.2511	0.1354	0.1031	0.0966
	GATE	0.2721	0.2528	0.1369	0.1030	0.0998
*C_35_H_54_N_6_O_2_*(2f)	XCOM	0.2921	0.2652	0.1445	0.1103	0.1032
	Phy-X	0.3017	0.2685	0.1448	0.1103	0.1033
	GATE	0.3027	0.2918	0.1531	0.1187	0.1072

**Table 2:** MIC values of compounds 2a–2f and antibiotics against microorganisms.

Microorganisms	Compounds						Antibiotics	

	2a	2b	2c	2d	2e	2f	Ampicillin	Fluconazole
*B*. *cereus*	625	625	625	625	1250	625	15	N.T
*B. subtilis*	625	625	625	312	625	312	10	N.T
*Y. pseudotuberculosis*	625	625	625	625	625	625	18	N.T
*K. pneumoniae*	625	625	625	312	1250	156	16	N.T
*E. coli*	625	625	625	625	1250	625	8	N.T
*P. aeruginosa*	625	625	625	625	625	625	128	N.T
*S. aureus*	625	312	625	625	1250	312	10	N.T
*E. faecalis*	625	625	625	625	625	625	10	N.T
*C. albicans*	156	312	156	78	1250	78	N.T	8

Minimal inhibition concentration (MIC) values were given as μg/mL. N.T: not tested.

**Table 3:** Binding energy, inhibition constant (K_i_), H bond count, and interacted residues of compounds 2d and 2f with receptor proteins.

Compound	Protein PDB ID	Binding Energy (kcal/mol)	K_i_ value	Number of HBs	Van-der Walls Interactions	Interacting Key Residues

2d	1HNJ	−8.91	296.19 nM	3	Thr80, Ala83, Ala86, Asp107, Val108, Leu142, Ile156, Phe157, Asn193, Leu205, Met207, Gly209, Asn210,, Val212, Phe213, Asn247, Ile250, Asn274, Phe304	Thr81, Ala109, Ala111, Cys112, Leu189, Thr190, Leu191, Ala246, Gly305, Gly306
	2VF5	−10.90	10.19 nM	4	Trp313, Ser316, Ile429, Asp432, Arg472, Gln475, His566, Val567, Glu569, Ala572, Tyr576,	Tyr312, Leu317, Gly473, Asp474, Ala520, Asp548, Val570
	5CDN	−7.51	3.12 μM	6	Ala94, Gln95, Asp96, Phe97, Tyr101, Val104, Asp105, Phe110, Thr129, Phe480, Asp482	Pro102, Gly106, Gly111, Lys130, Ile131, Arg479, Gly481, Asp483
	5MMN	−9.08	221.83 nM	4	Ala47, Glu50, Gly77, Ile78, Pro79, Val97, Leu98, Val118, Gly119, Thr165	Glu42, Asp45, Asn46, Asp73, Ile94, Val120, Ser121

2f	1HNJ	−7.92	1.55 μM	4	Trp32, Thr37, Ile156, Leu189, Thr190, Leu205, Met207, Gly209, Asn210, Phe213, His244, Asn274, Se276, Gly306,	Arg36, Thr81, Ala111, Cys112, Leu142, Phe157, Leu191, Asn193, Val212, Ala246, Ile250, Phe304, Gly305
	2VF5	−9.26	162.93 nM	2	Tyr312, Trp313, Ser316, Leu317, His435, Gly473, Gln475, Pro521, Asn522, Leu525, His566, Val567, Glu568, Val570,	Asp432, Arg472, Asp474, Ala520, Asp548, Glu569
	5CDN	−8.21	964.45 nM	3	Ala94, Gln95, Val104, Gly108, Asn109, Thr129, Phe110, Arg479, Phe480, Asp482, Asp483	Tyr101, Pro102, Asp105, Gly106, Ile131, Gly481
	5MMN	−8.22	945.54 nM	3	Asn46, Ala47, Ile48, Glu50, Gly75Arg76, Gly77, Pro79, Val97, Leu98, Val118, Gly119, Ser121, Thr165	Asp45, Asp46, Asp73, Ile78, Ile94, Val120,

**Table 4:** MM/PBSA-derived binding free energy components for the 2VF5–2d complex.

Compound	MM-PBSA (kcal/mol ± SD)				
	Δ*G*_vdW_ (van der Waals)	Δ*G*_ele_ (Electrostatic)	Δ*G*_solv, GB_ (Polar solvation)	Δ*G*_nonpolar_ (Non-polar Solvation)	Δ*G*_bind_ (Total Binding)
Complex	−42.73 ± 0.61	−22.59± 3.87	41.99 ± 0.95	−3.98 ± 0.04	−27.31 ± 1.90

ΔG_vdW_: van der Waals interaction energy; ΔG_ele_: electrostatic interaction energy; ΔG_solv_: polar solvation energy (Generalized Born model); ΔG_nonpolar_: nonpolar solvation energy (SASA-derived); ΔG_bind_: total binding free energy.

## Data Availability

All data are available in the manuscript.

## Footnotes

1 PDB(2025). Protein Data Bank [online]. Website https://www.rcsb.org/ [accessed 12 June 2025]
