# Supplementary figures and images for: New bis-piperazine derivatives: synthesis, characterization (IR, NMR), gamma-ray absorption, antimicrobial activity, molecular docking and dynamics study
Source: Turk J Chem. 2025 Aug 16;49(6):736–53. doi: 10.55730/1300-0527.3767 (PMC12779072; doi:10.55730/1300-0527.3767)

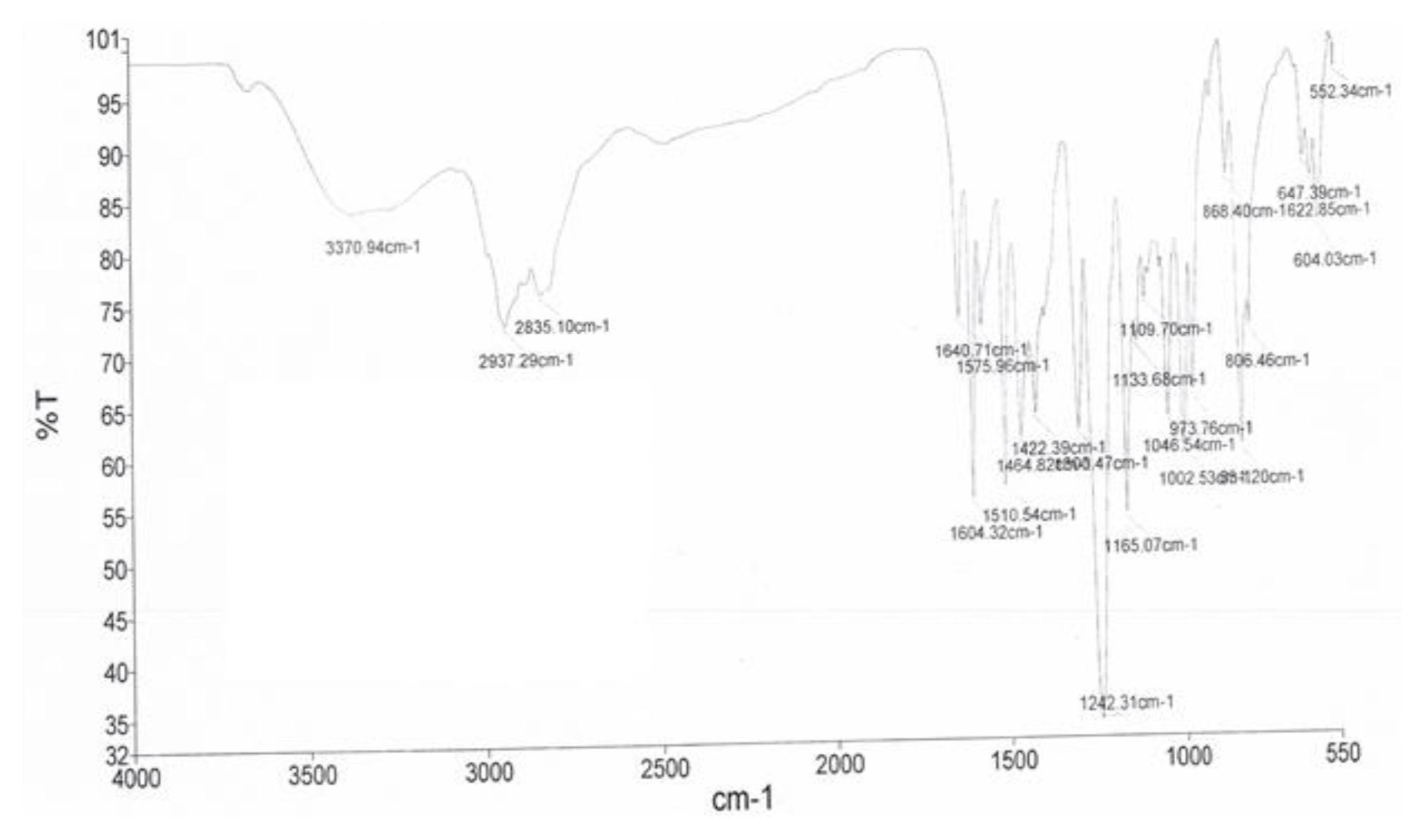

Supplement: Figure S1 — IR spectrum of compound 2a. [file tjc-49-06-736s1.tif]

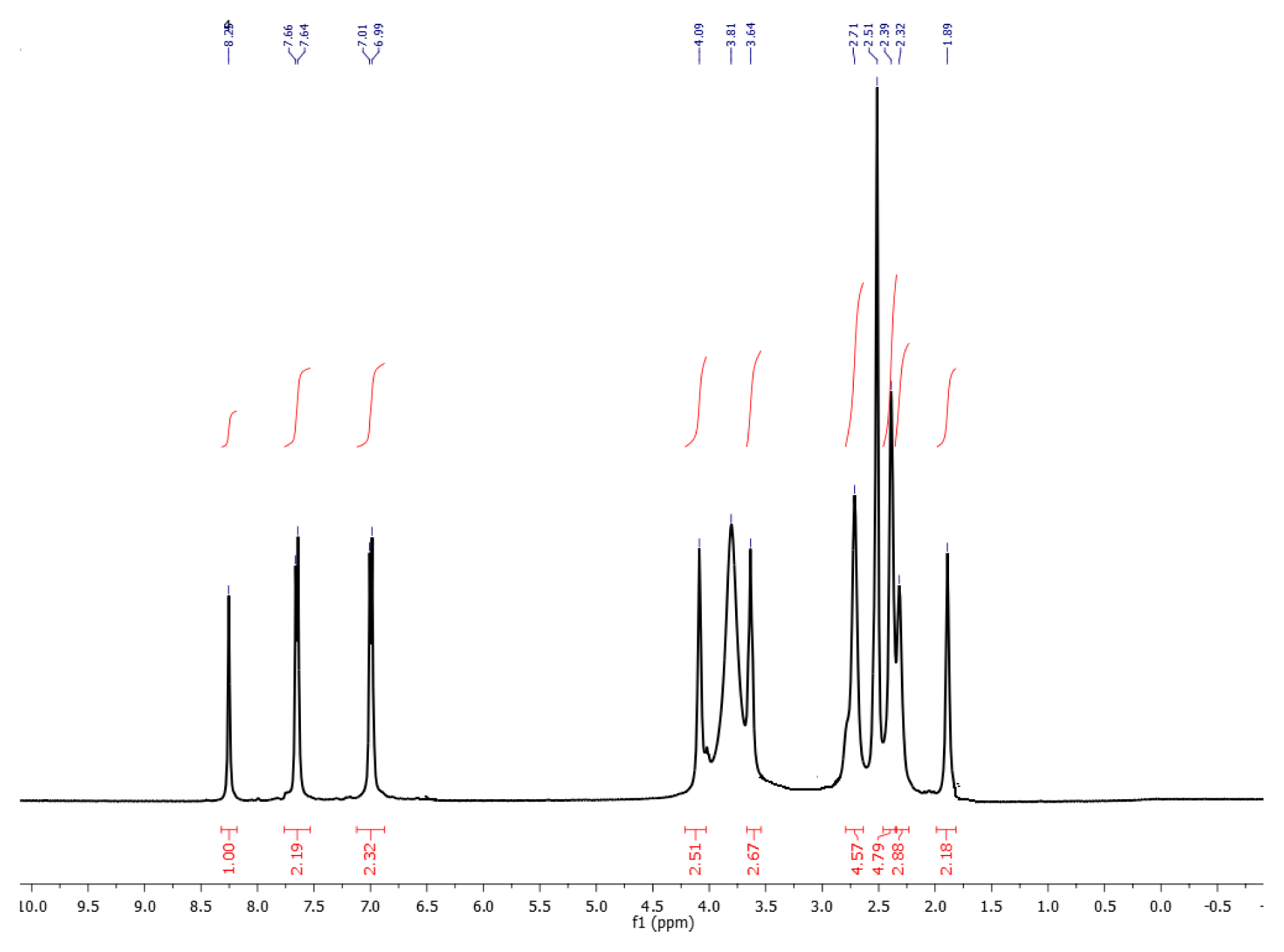

Supplement: Figure S2 — 1H-NMR spectrum of compound 2a. [file tjc-49-06-736s2.tif]

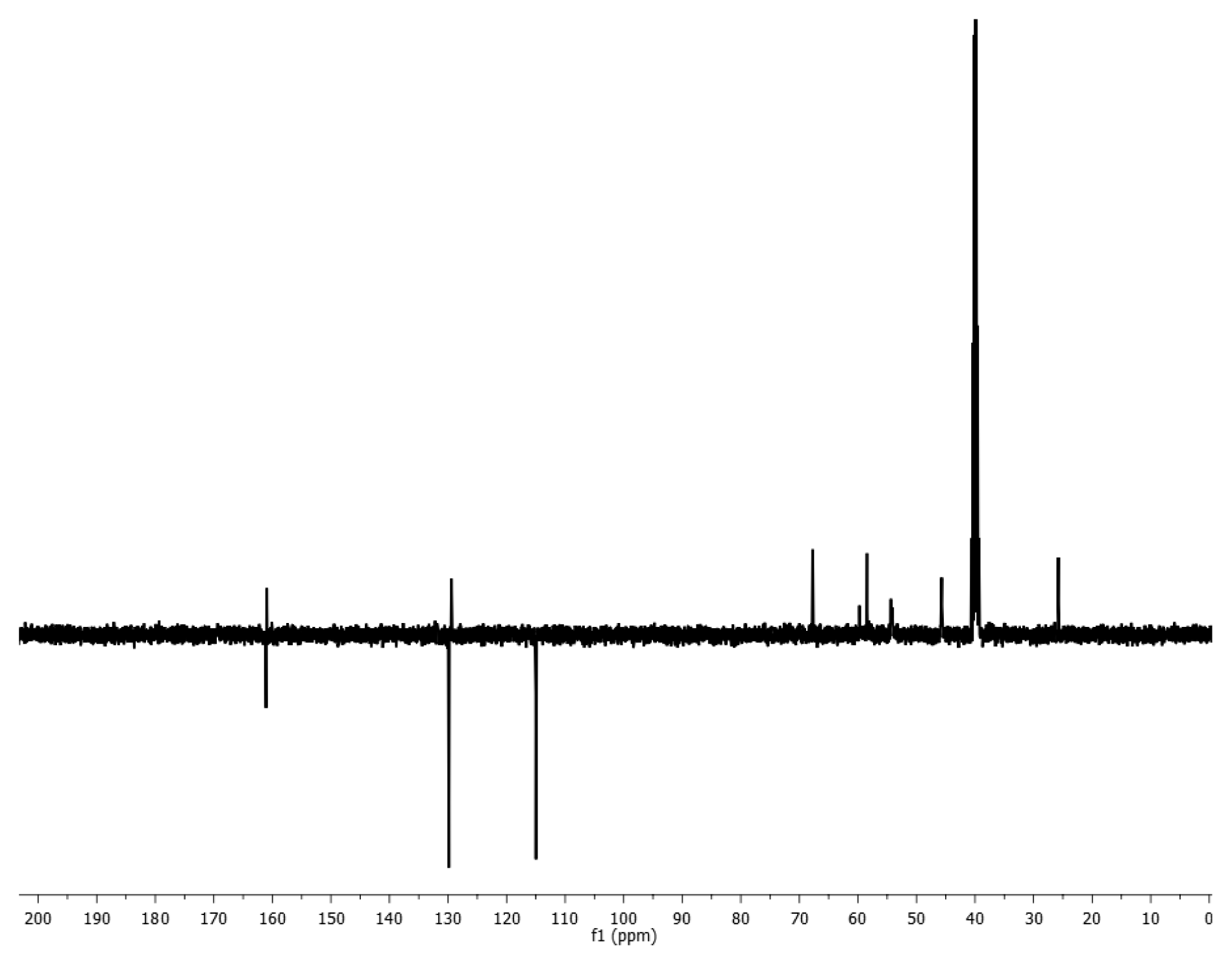

Supplement: Figure S3 — 13C-NMR (APT) spectrum of compound 2a. [file tjc-49-06-736s3.tif]

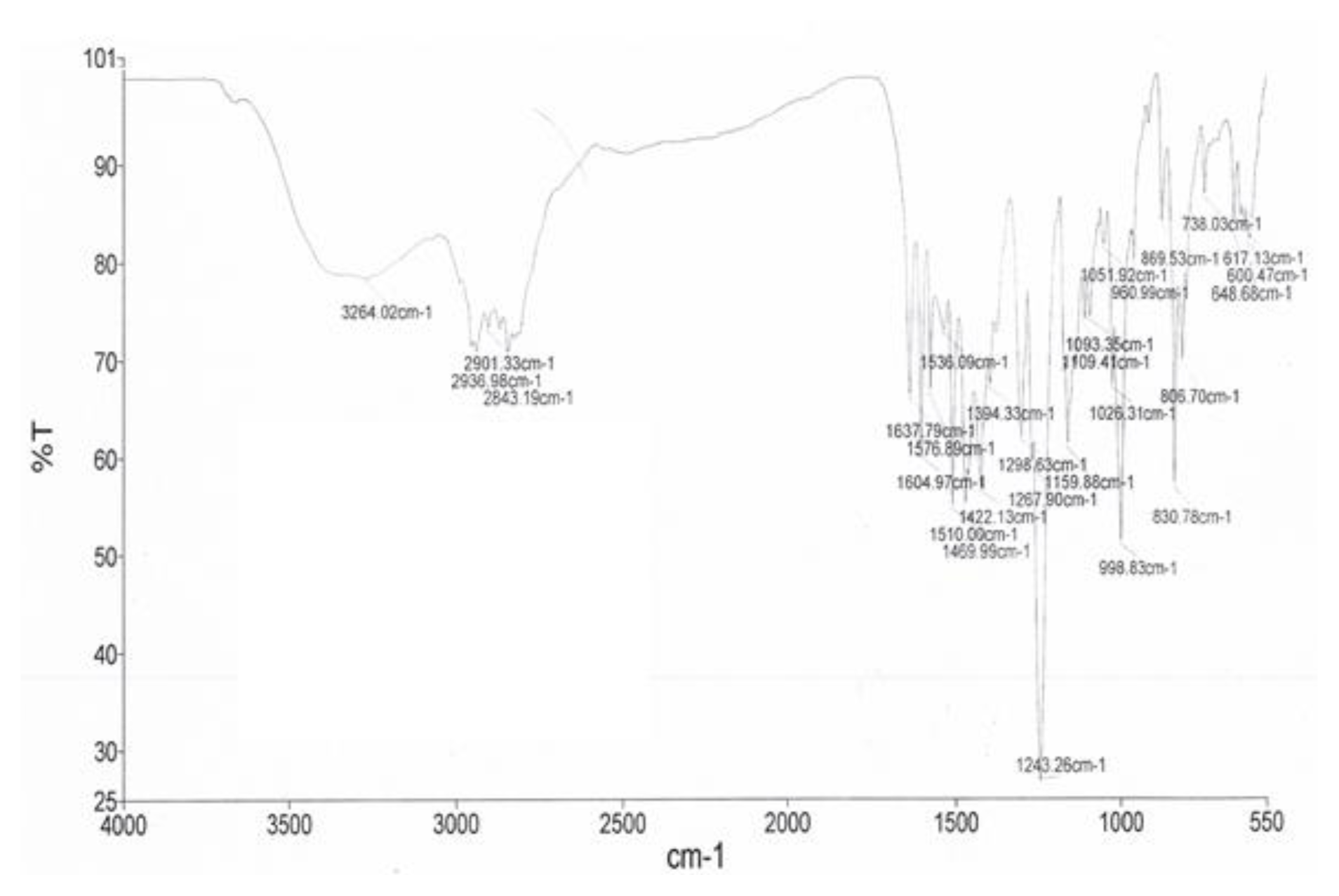

Supplement: Figure S4 — IR spectrum of compound 2b. [file tjc-49-06-736s4.tif]

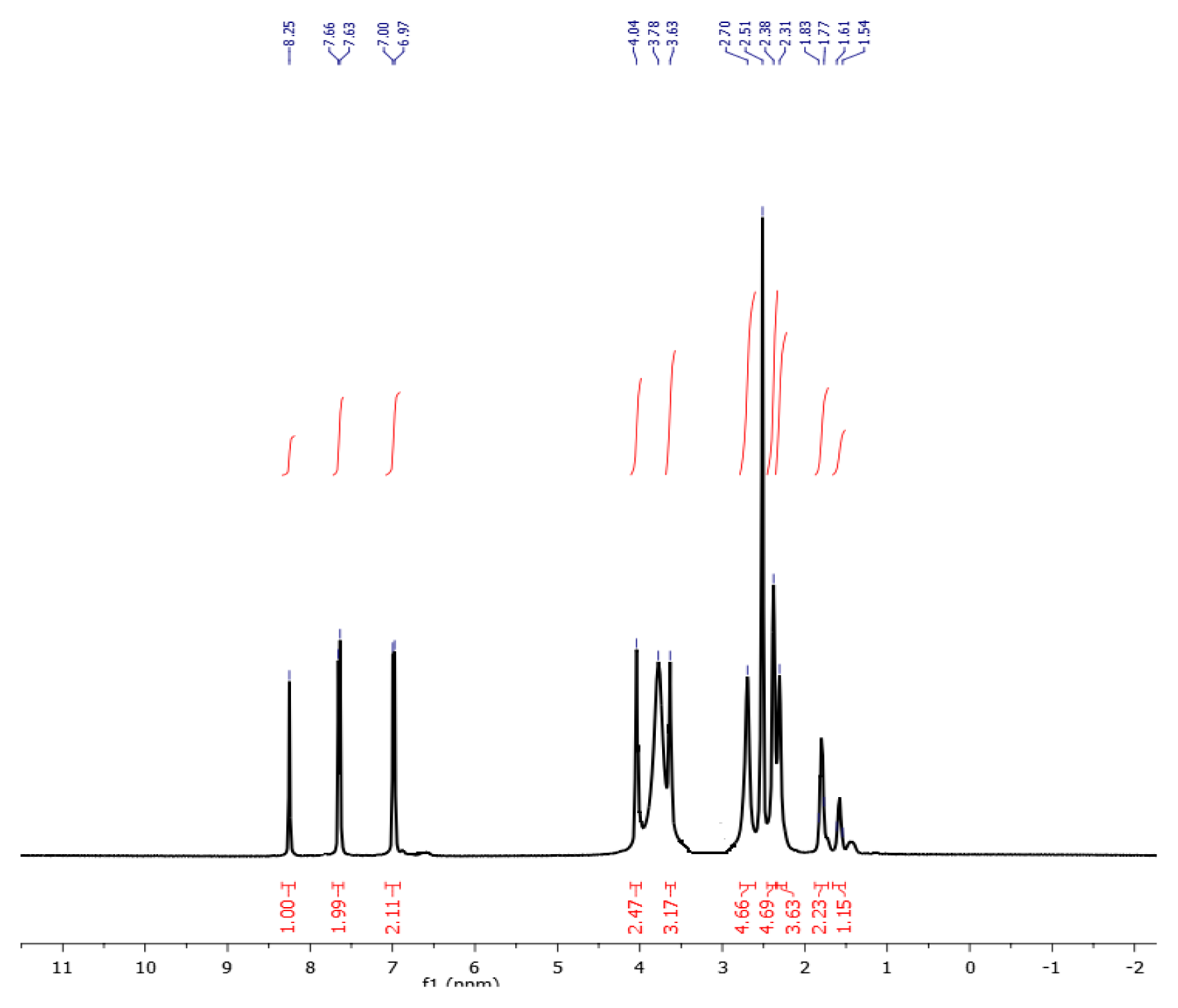

Supplement: Figure S5 — 1H-NMR spectrum of compound 2b. [file tjc-49-06-736s5.tif]

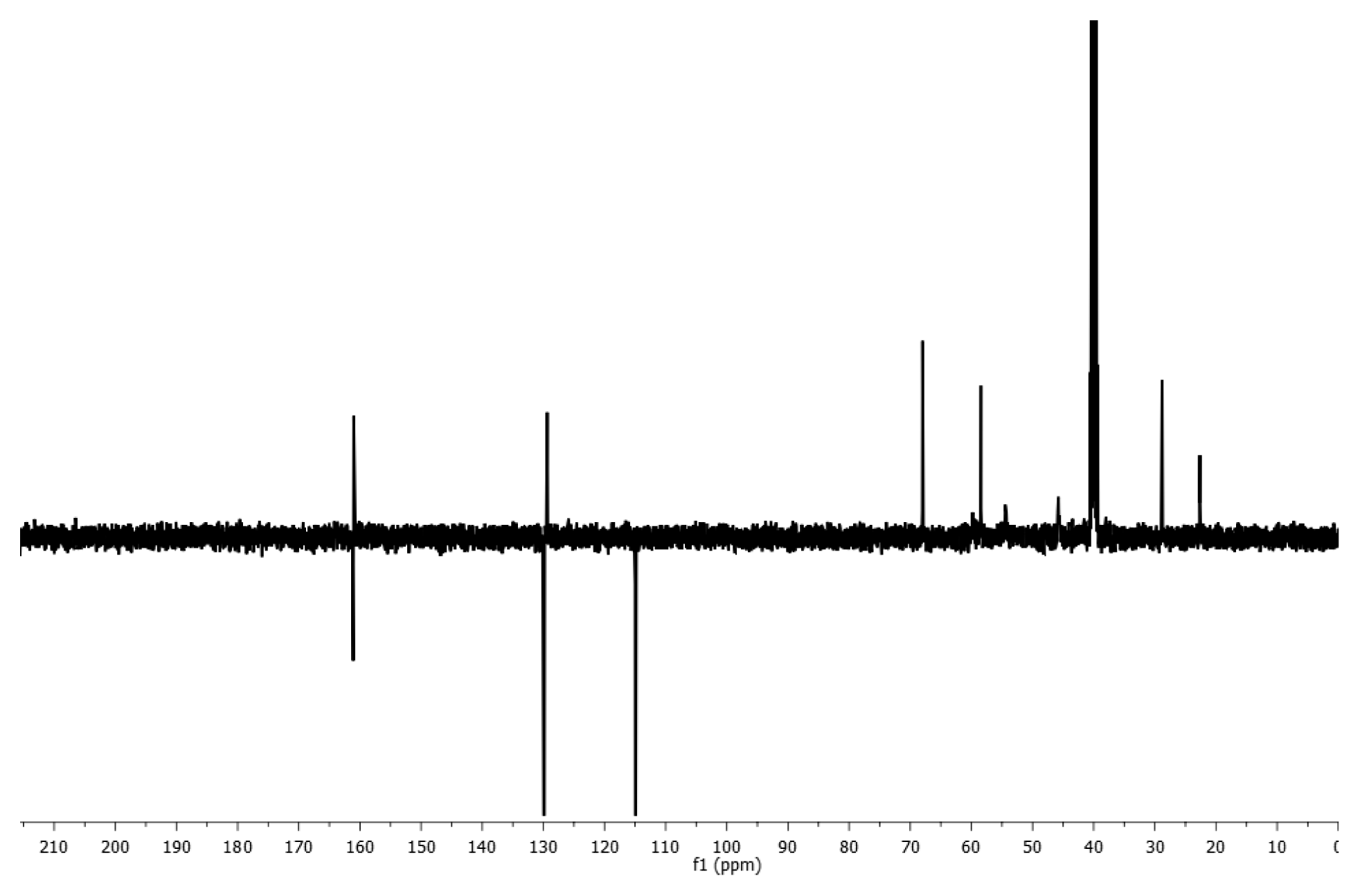

Supplement: Figure S6 — 13C-NMR (APT) spectrum of compound 2b. [file tjc-49-06-736s6.tif]

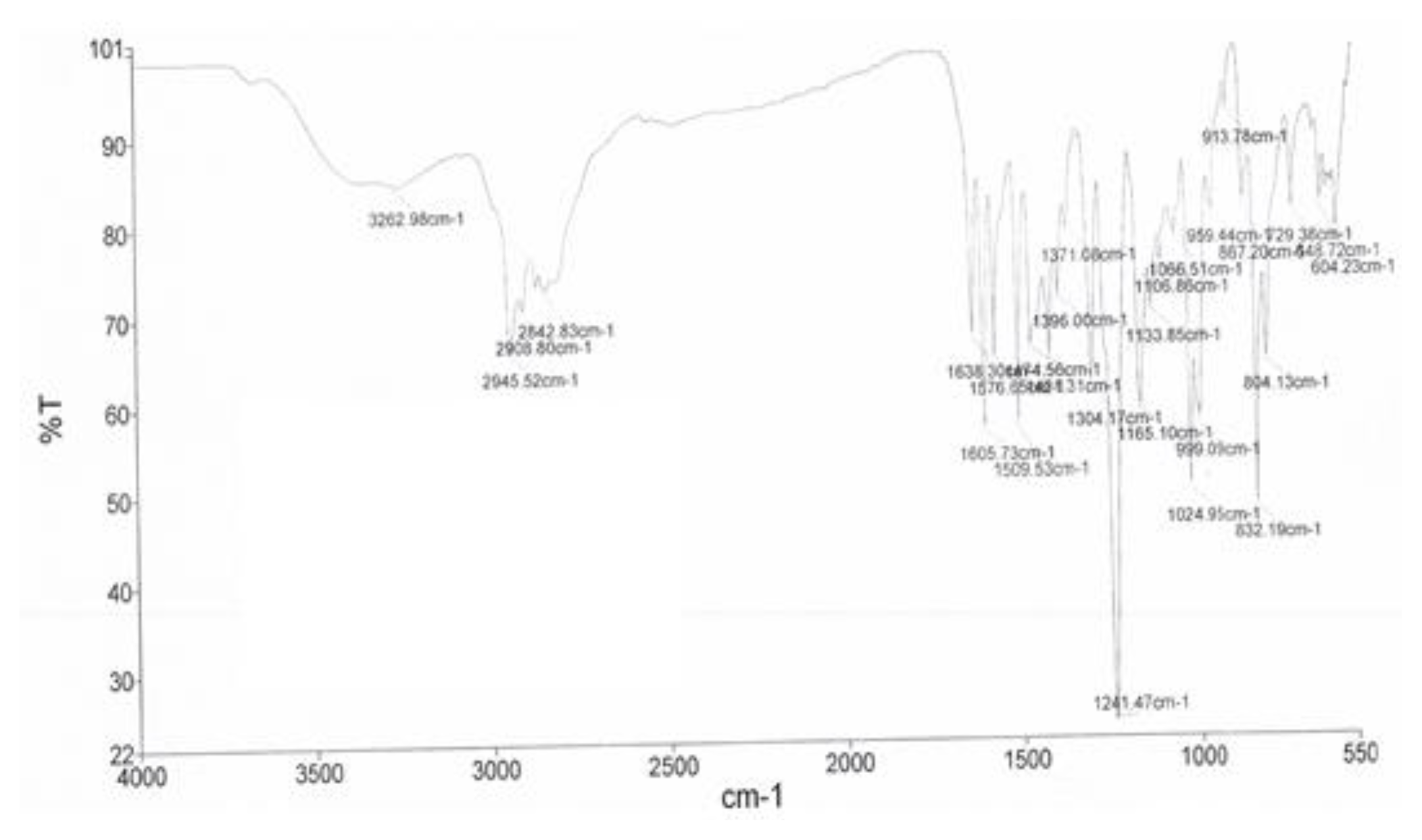

Supplement: Figure S7 — IR spectrum of compound 2c. [file tjc-49-06-736s7.tif]

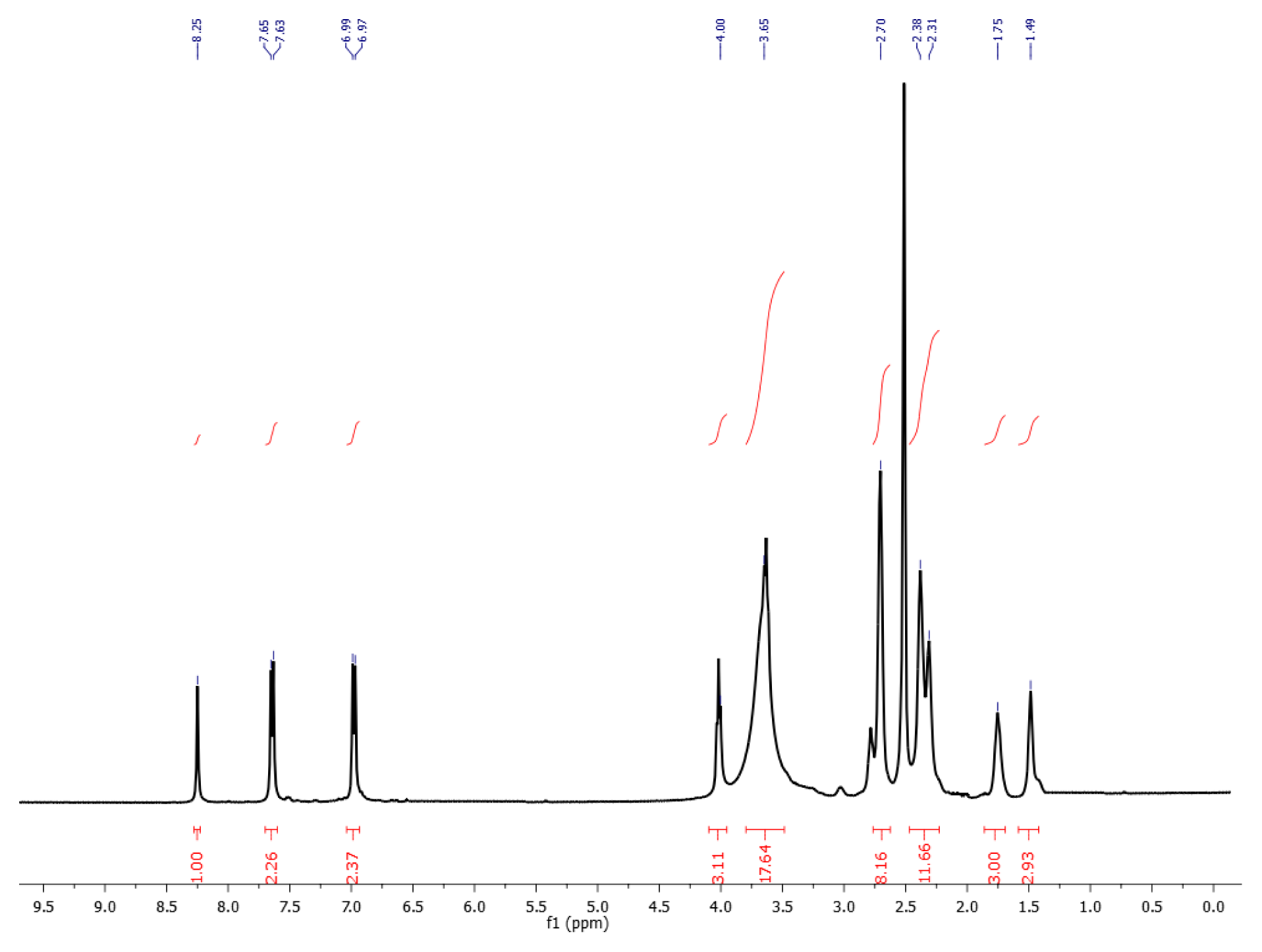

Supplement: Figure S8 — 1H-NMR spectrum of compound 2c. [file tjc-49-06-736s8.tif]

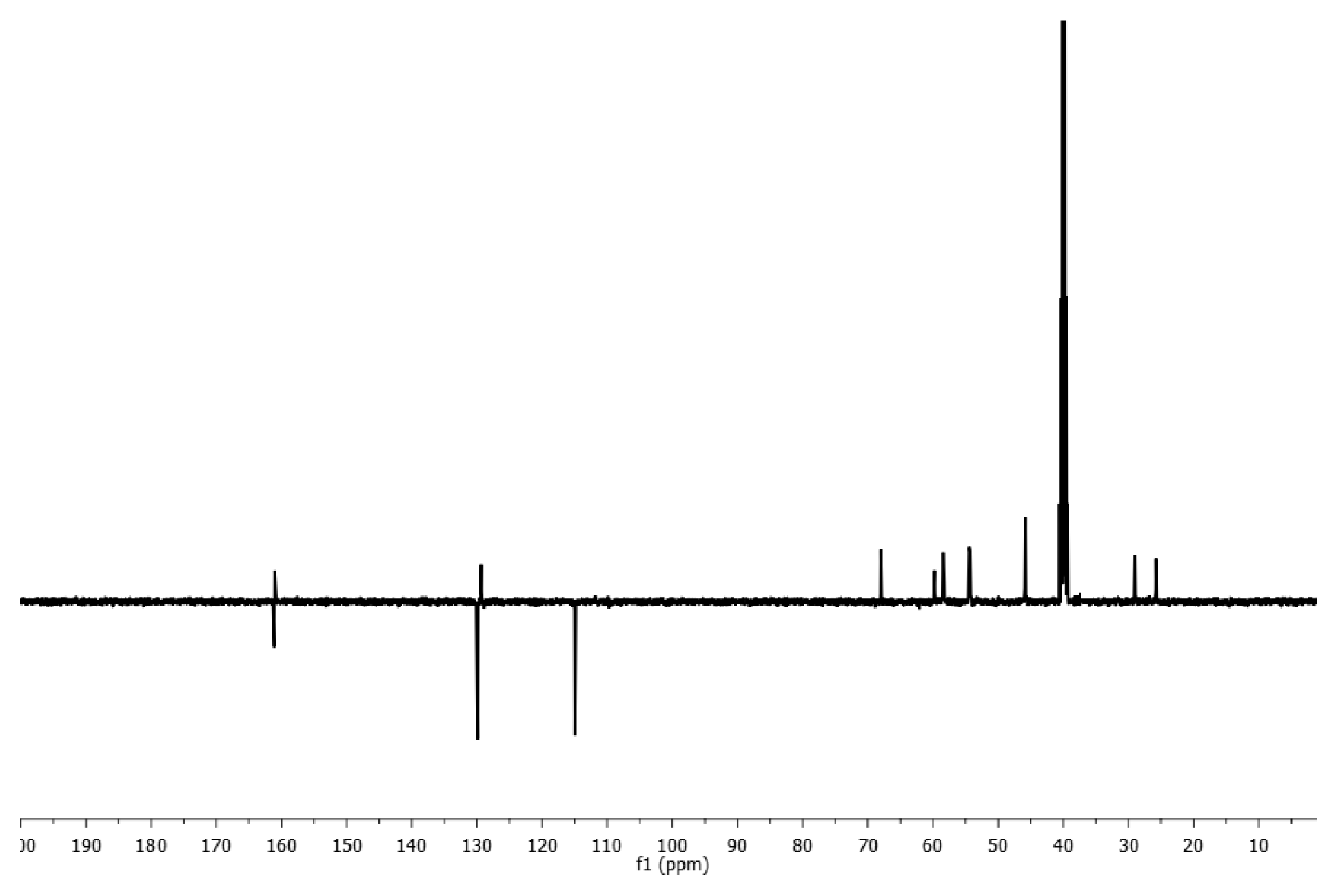

Supplement: Figure S9 — 13C-NMR (APT) spectrum of compound 2c. [file tjc-49-06-736s9.tif]

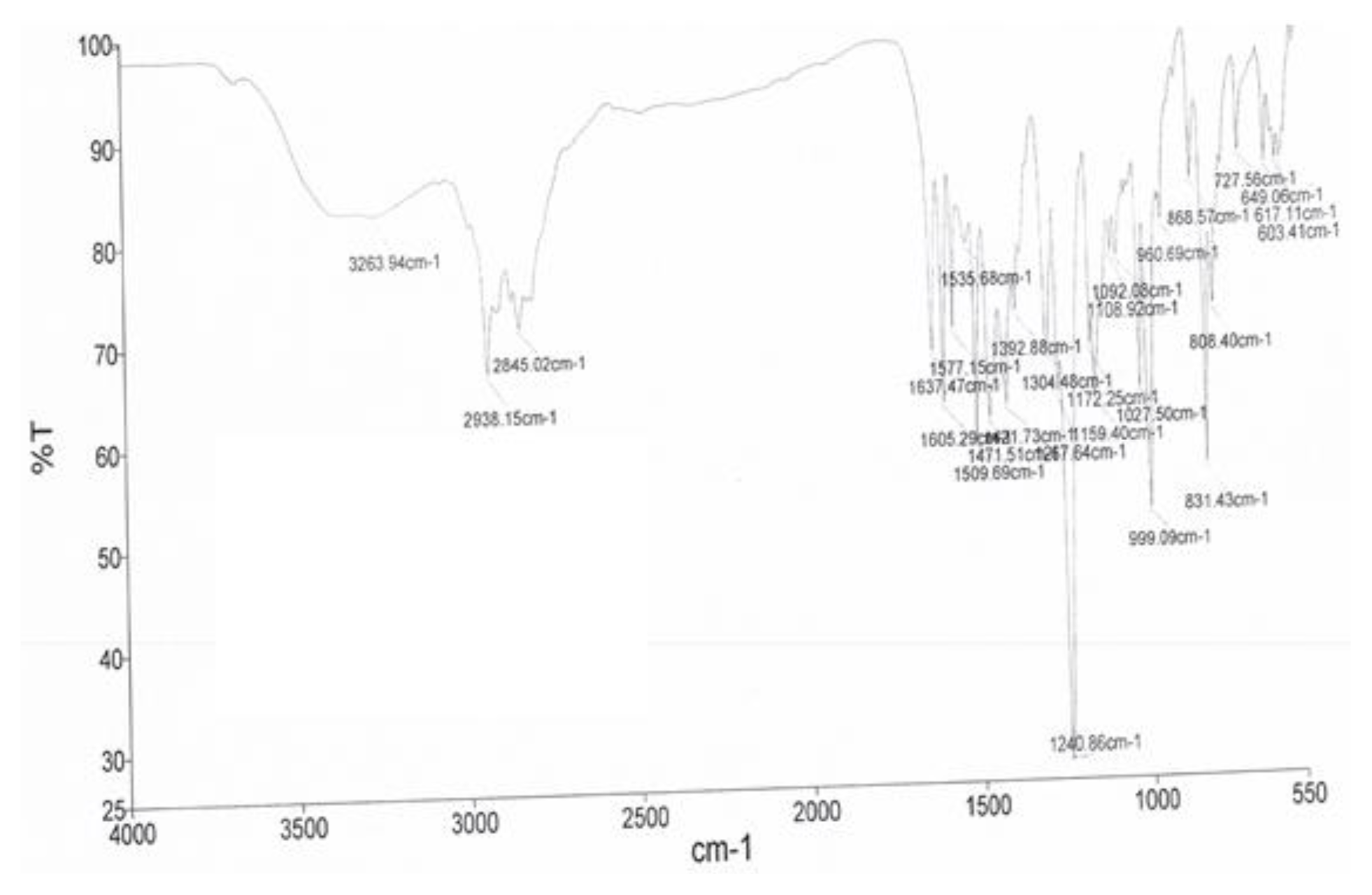

Supplement: Figure S10 — IR spectrum of compound 2d. [file tjc-49-06-736s10.tif]

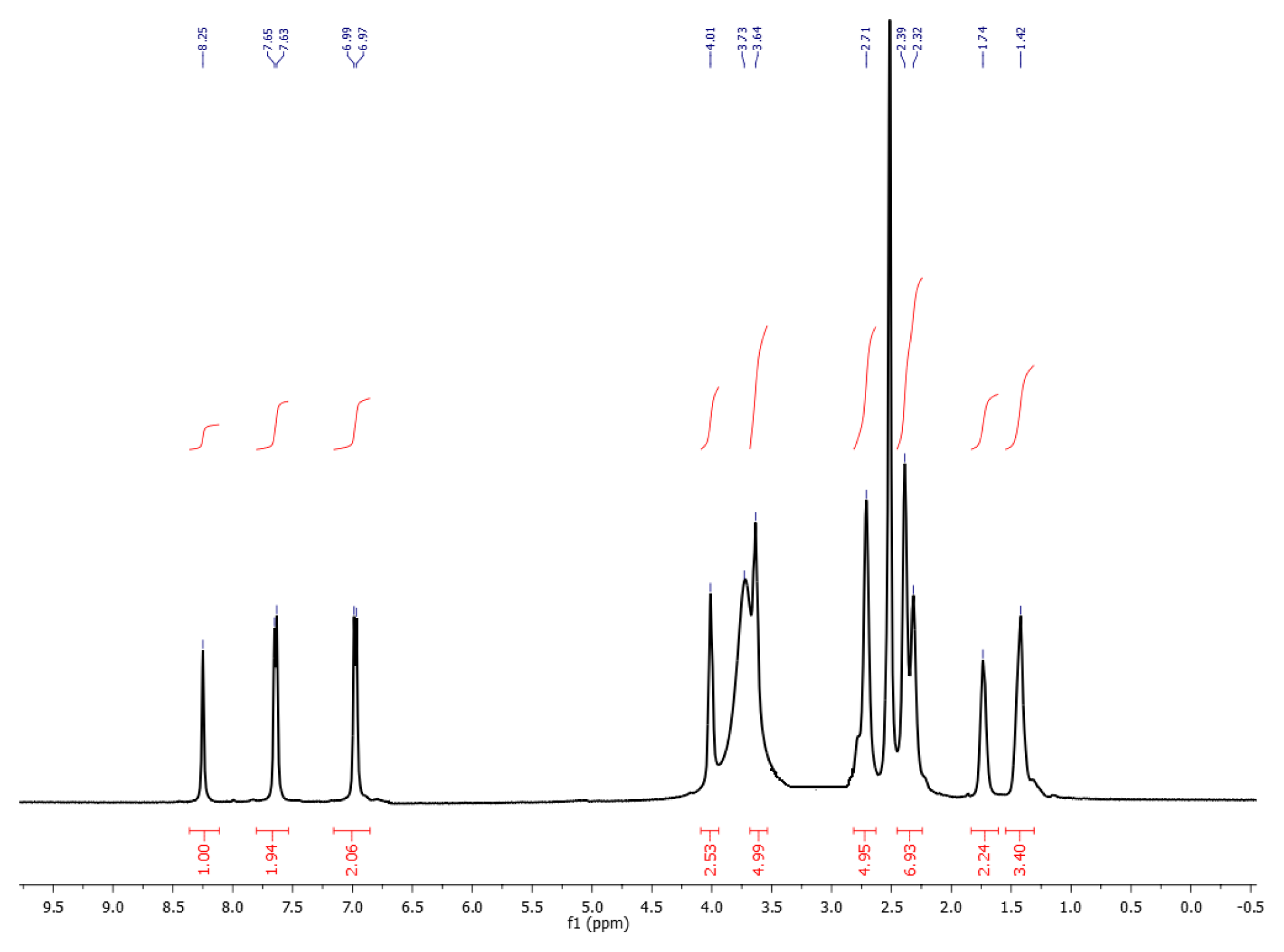

Supplement: Figure S11 — 1H-NMR spectrum of compound 2d. [file tjc-49-06-736s11.tif]

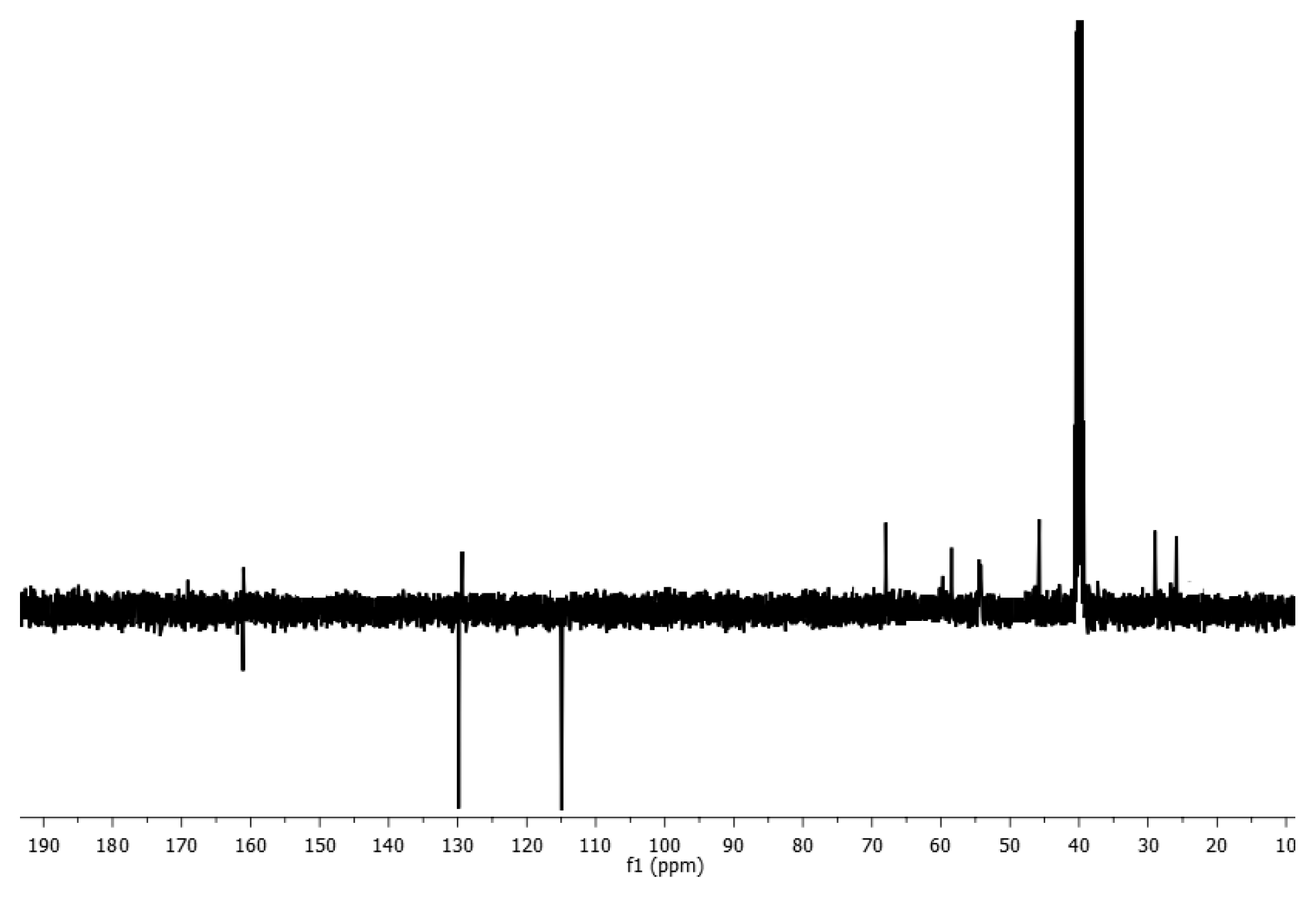

Supplement: Figure S12 — 13C-NMR (APT) spectrum of compound 2d. [file tjc-49-06-736s12.tif]

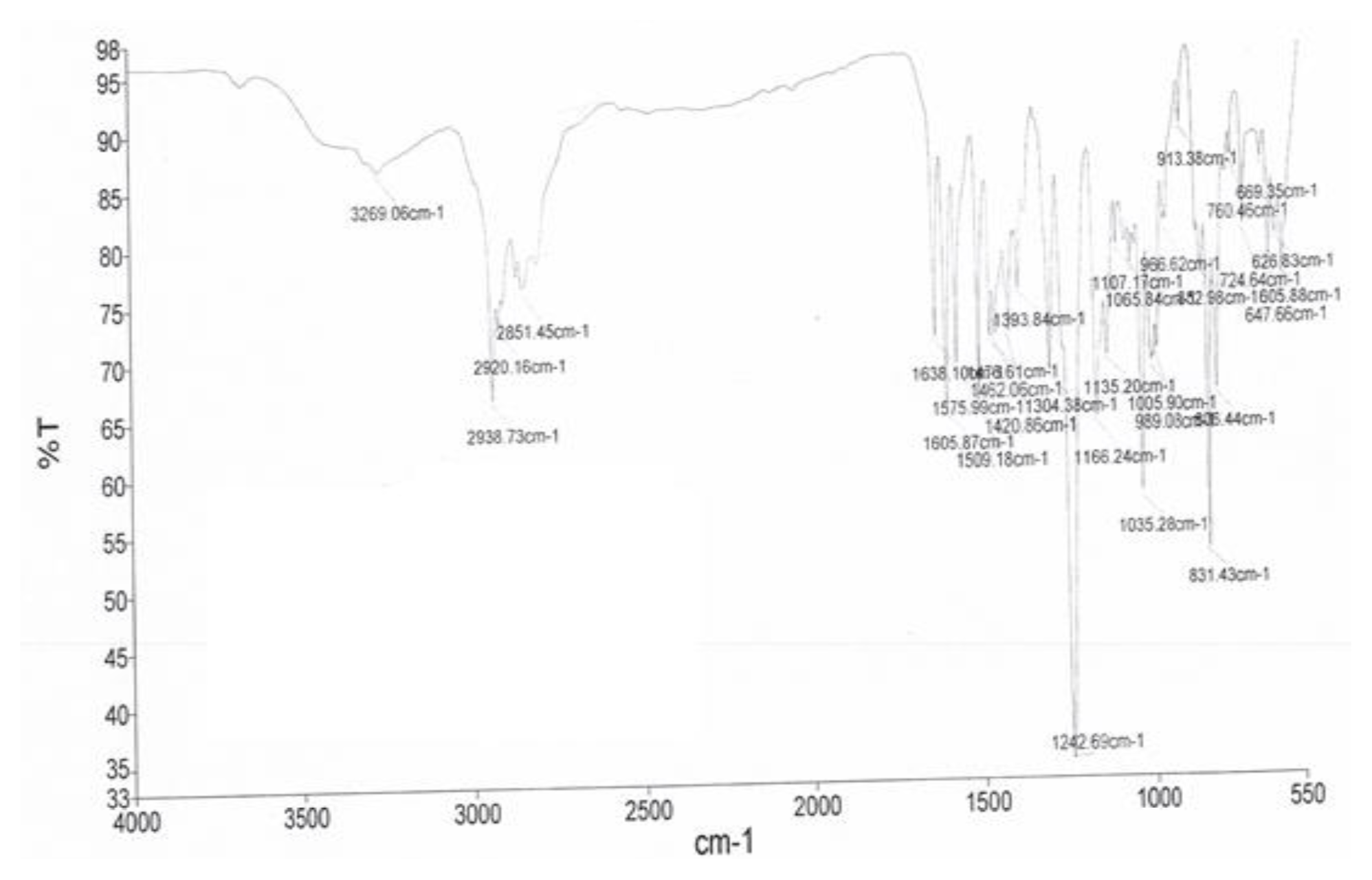

Supplement: Figure S13 — IR spectrum of compound 2e. [file tjc-49-06-736s13.tif]

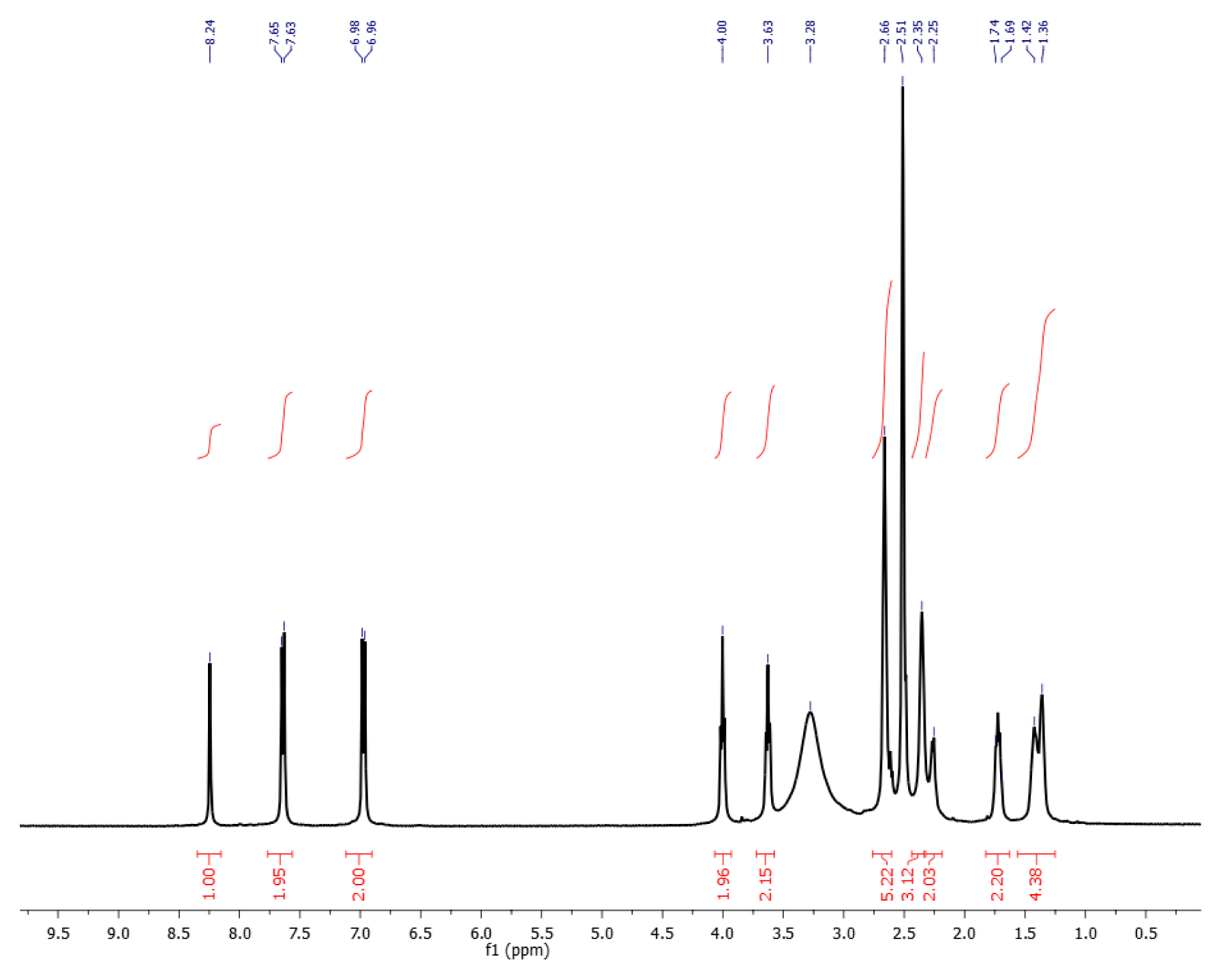

Supplement: Figure S14 — 1H-NMR spectrum of compound 2e. [file tjc-49-06-736s14.tif]

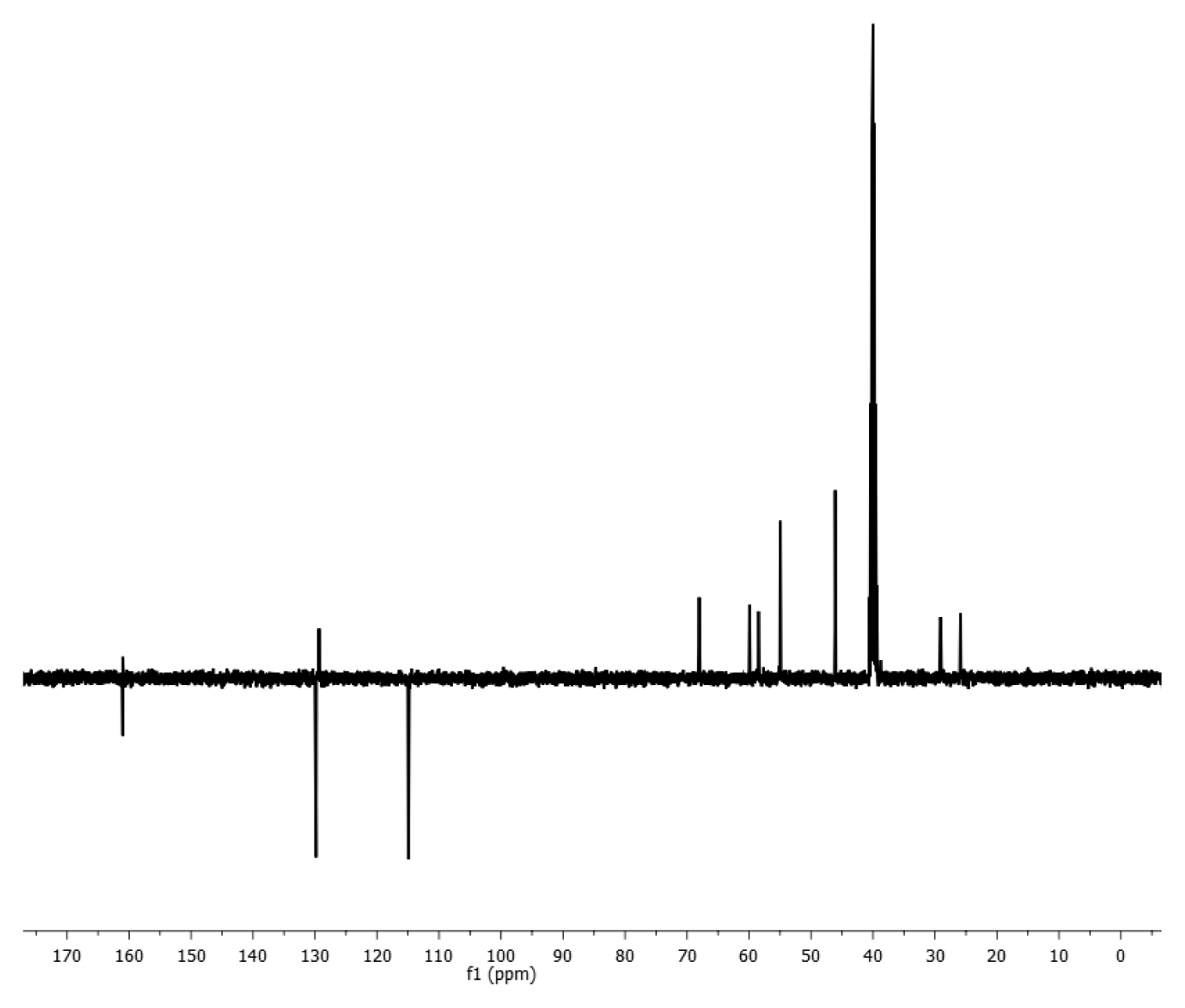

Supplement: Figure S15 — 13C-NMR (APT) spectrum of compound 2e. [file tjc-49-06-736s15.tif]

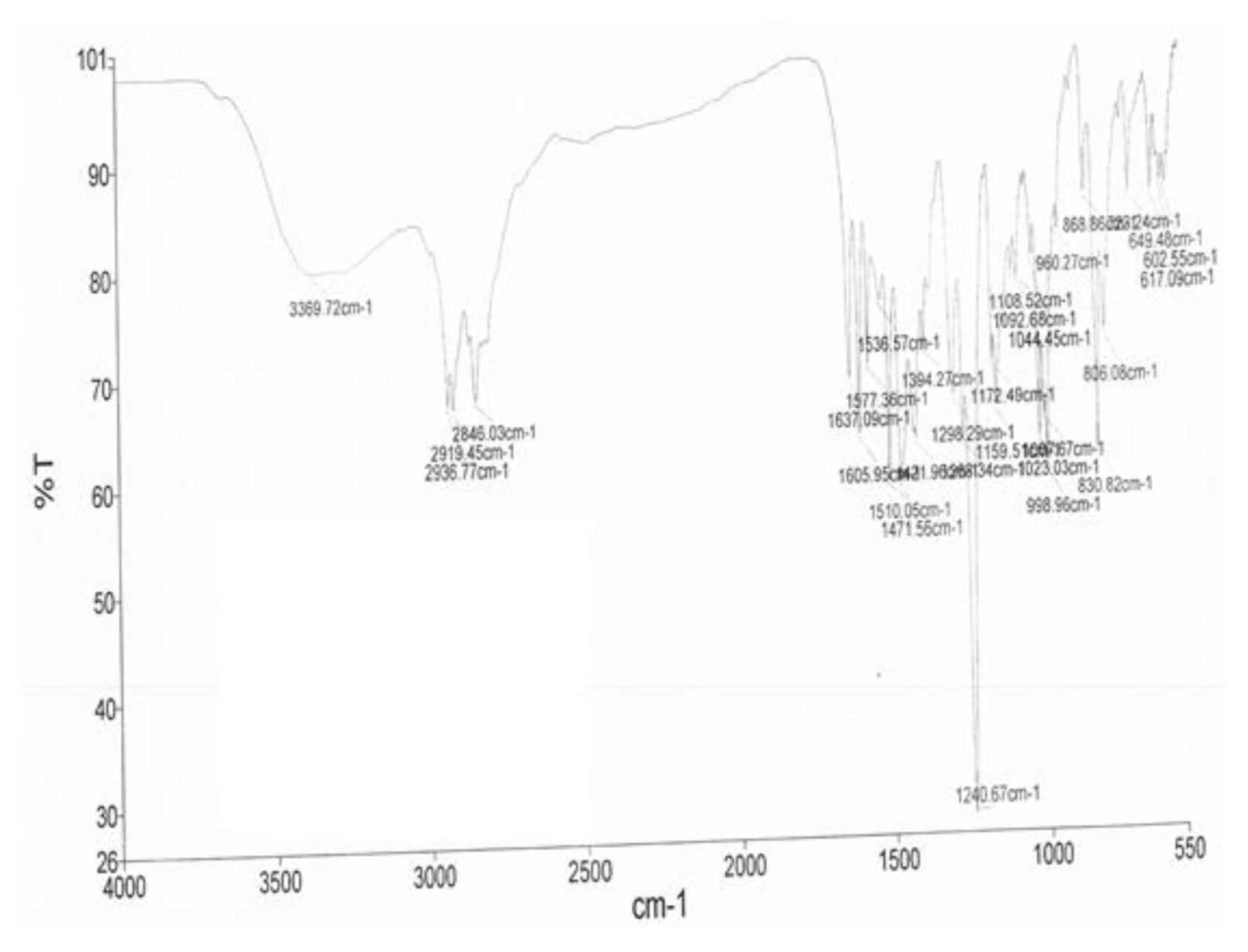

Supplement: Figure S16 — IR spectrum of compound 2f. [file tjc-49-06-736s16.tif]

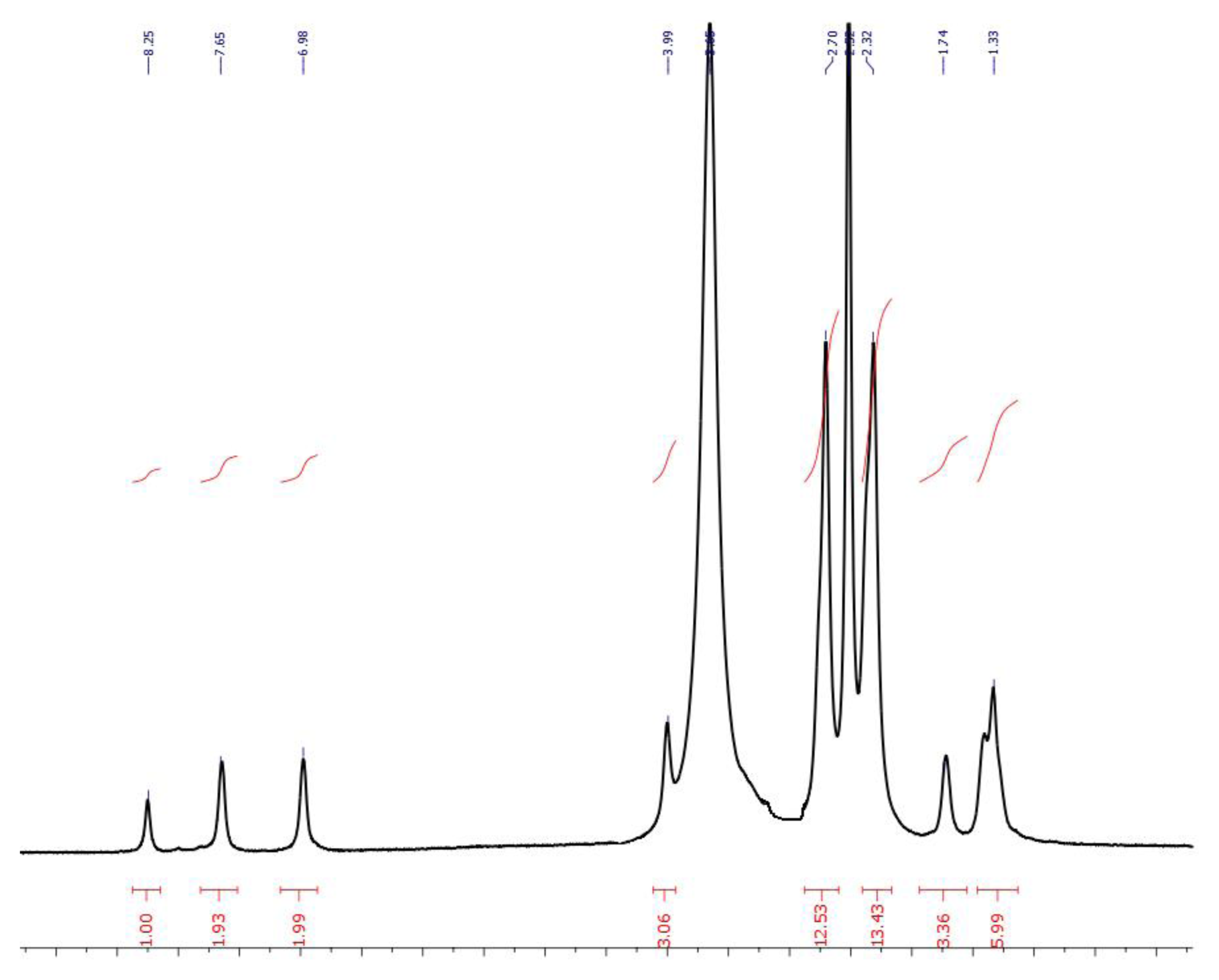

Supplement: Figure S17 — 1H-NMR spectrum of compound 2f. [file tjc-49-06-736s17.tif]

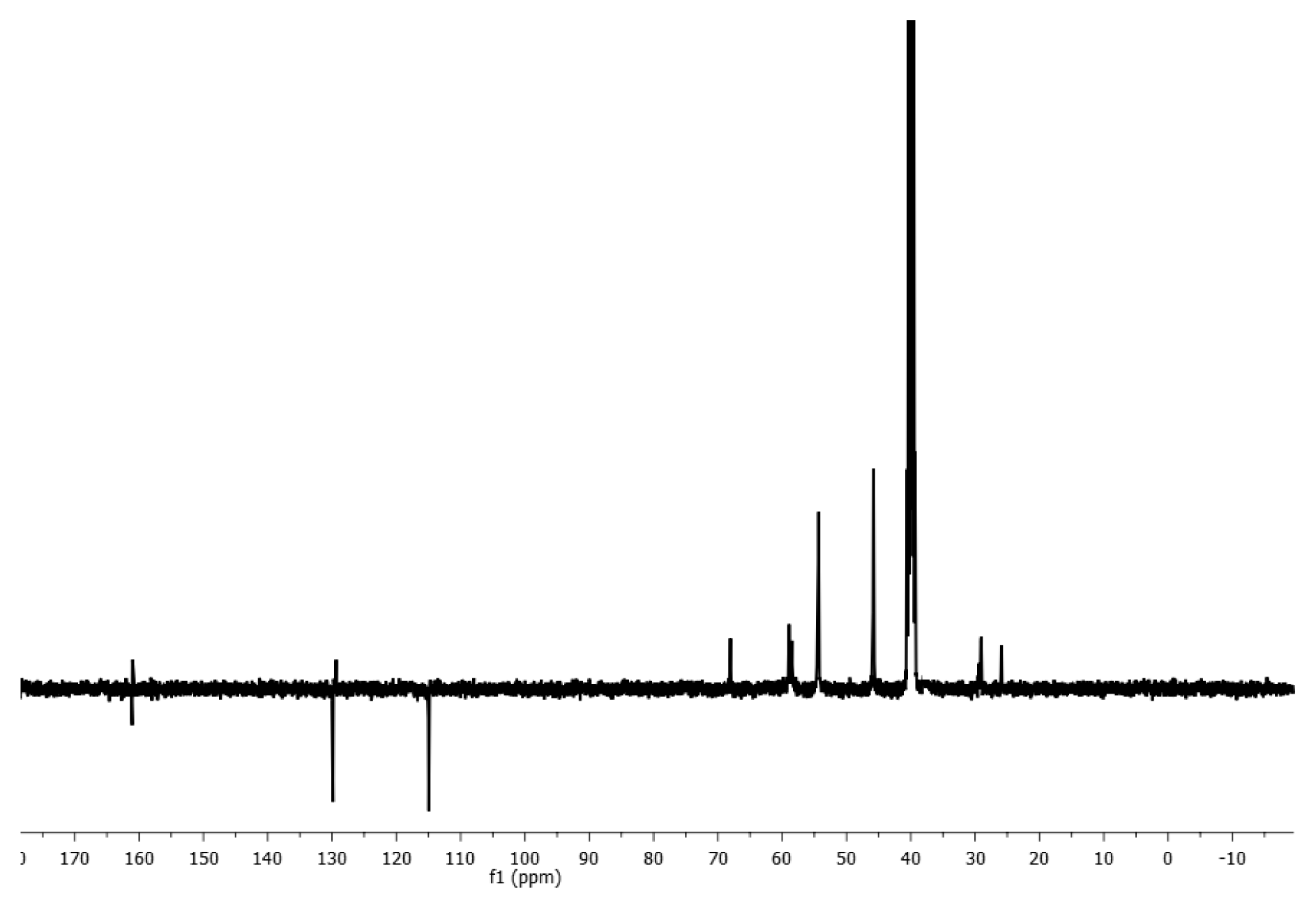

Supplement: Figure S18 — 13C-NMR (APT) spectrum of compound 2f. [file tjc-49-06-736s18.tif]
